# Identification and characterization of novel *ETV4* splice variants in prostate cancer

**DOI:** 10.1038/s41598-023-29484-1

**Published:** 2023-03-31

**Authors:** Irene Cosi, Annalisa Moccia, Chiara Pescucci, Uday Munagala, Salvatore Di Giorgio, Irene Sineo, Silvestro G. Conticello, Rosario Notaro, Maria De Angioletti

**Affiliations:** 1 Core Research Laboratory, Istituto per lo Studio, la Prevenzione e la Rete Oncologica (ISPRO), Viale Pieraccini 6, 50139 Florence, Italy; 2 ICCOM - National Research Council, Sesto Fiorentino, Florence, Italy; 3 IFC - National Research Council, Pisa, Italy

**Keywords:** Cancer, Cell biology, Molecular biology, Molecular medicine, Oncology

## Abstract

ETV4, one of ETS proteins overexpressed in prostate cancer, promotes migration, invasion, and proliferation in prostate cells. This study identifies a series of previously unknown *ETV4* alternatively spliced transcripts in human prostate cell lines. Their expression has been validated using several unbiased techniques, including Nanopore sequencing. Most of these transcripts originate from an in-frame exon skipping and, thus, are expected to be translated into ETV4 protein isoforms. Functional analysis of the most abundant among these isoforms shows that they still bear an activity, namely a reduced ability to promote proliferation and a residual ability to regulate the transcription of ETV4 target genes. Alternatively spliced genes are common in cancer cells: an analysis of the TCGA dataset confirms the abundance of these novel *ETV4* transcripts in prostate tumors, in contrast to peritumoral tissues. Since none of their translated isoforms have acquired a higher oncogenic potential, such abundance is likely to reflect the tumor deranged splicing machinery. However, it is also possible that their interaction with the canonical variants may contribute to the biology and the clinics of prostate cancer. Further investigations are needed to elucidate the biological role of these *ETV4* transcripts and of their putative isoforms.

## Introduction

Erythroblast Transformation Specific (ETS) proteins are a family of transcription factors, which can act as activators or repressors of their gene targets^1^. ETS proteins are involved in the regulation of various signaling cascades, which control diverse biological processes: proliferation, differentiation, development, transformation, apoptosis, migration, invasion, and angiogenesis^2^**.** These proteins are characterized by the presence of the ETS domain, a highly conserved DNA-binding domain that recognizes the purine-rich DNA motif 5’-GGA(A/T)-3′ ^3–5^. In addition to the ETS domain, these proteins contain a variety of diverse regulatory domains that characterize different subfamilies^1^. For example, the PEA3 subfamily, which includes the ETS translocation variant 4 (*ETV4*) gene, is characterized by two acidic domains, each one constituting the core of a transcriptional activation domain: the 32 aa acidic domain near the amino terminus is more potent than the 60 aa acidic domain near at the carboxyl terminus^6–8^. Moreover, the activity of the ETS domain and the activation domains are regulated by their flanking sequences^6–9^.

Aberrant expression of ETS genes is present in several types of cancer^10^. In prostate cancer, this is a frequent and -usually- early phenomenon^11–13^, which results from a chromosomal translocation involving any of several ETS genes^14–16^. The ETS genes most frequently involved in these translocations are *ERG*, *ETV1*, *ETV4,* and *ETV5*, with *ERG* being by large the most common^13,14^.

*ETV4* (also called *PEA3* or *E1AF*) is a member of the ETS transcription factor family, belonging to the PEA3 subfamily. In humans, the *ETV4* gene maps to the long arm of chromosome 17 (band 17q21), and it consists of 13 exons encoding for 9 transcripts, which differ in the 5' UTR and the usage of different start codons^17^. *ETV4* is expressed during embryogenesis and it contributes to motor axon guidance^18^. Furthermore, it is involved in developmental processes such as branching morphogenesis (mammary gland, lung, and kidney)^19–21^ and the formation of limb buds^22^. *ETV4* overexpression has been correlated with the activation of cancer-related genes relevant to cell proliferation and to invasiveness^23–26^. Aberrant expression of *ETV4* has been observed in several cancers (breast, prostate, ovary, lung, and gastrointestinal tract cancer)^24,27,28^, and it has been associated with metastatic disease and with poor prognosis^29^**.**

In prostate cancer, *ETV4* is one of the ETS genes involved in chromosomal translocations^30–32^. Several studies in prostate cell lines have shown that *ETV4* induces many neoplastic features: invasiveness, cell migration^33,34^, cell cycle progression, anchorage-independent growth, tumor growth in a xenograft model and also epithelial mesenchymal transition^34^. In addition, prostate expression of *ETV4* results in the late development of mouse prostatic intraepithelial neoplasia in transgenic mice^35^ whereas occurrence of metastasis was associated with the late increase of *ETV4* expression in a mouse model with several genetic alterations (*pTen* loss, *NKX3.1* deletion, and a *KRAS* activating mutation) ^36^.

Alternative splicing is a process that generates multiple mRNA transcripts from a single gene, thus expanding the proteome^37,38^. Alternative splicing may rearrange the regulatory domains of several ETS genes to generate multiple isoforms with possible diverse functions^39–41^. Alternative splicing is a tightly regulated process that plays a relevant role in development and physiology^42,43^: in fact, its deregulation has been associated with a variety of diseases^43–46^**.** Recently, alternative splicing has been also included among the ‘hallmarks’ of cancer cells^47^. Alternative splicing has been described also in various genes that play a role in prostate cancer^48–50^, including members of the ETS gene family^39^. Multiple isoforms with distinctive oncogenic potential have been described for ERG^51–53^, ETV1^39^ and ETS1^54,55^, while data on ETV4 are not yet available.

We have investigated the expression of the alternative *ETV4* transcripts in prostate cell lines and have found a number of previously unreported *ETV4* transcripts. Furthermore, we analyzed the role of these *ETV4* transcripts and studied the effects of their putative isoforms on some phenotypic neoplastic features.

## Results

### Identification of novel alternative ETV4 transcripts in cell lines

**Figure 1:**
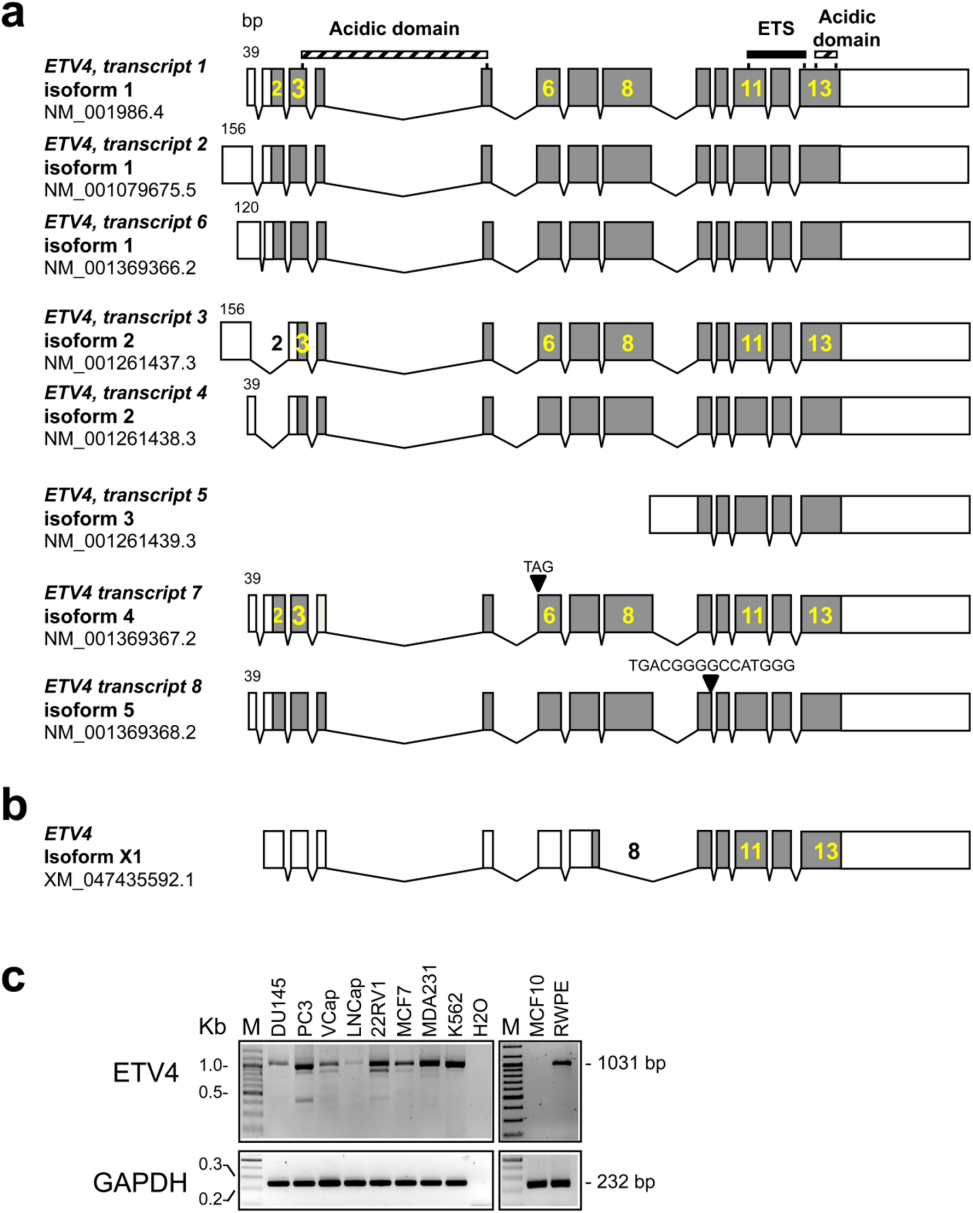
Schematic representation of the 9 transcripts of *ETV4* gene reported in the NCBI RefSeq database (https://www.ncbi.nlm.nih.gov/gene) according to the *ETV4* genomic sequence NC_000017.11 based on the GRCh38.p13 Primary Assembly. The identification numbers for each transcript and each protein isoform are reported. The position of functional features is shown on the top: DNA-binding ETS motif (black bar); Acidic domain (hatched bars). The exonic structure is shown with coding exon sequences in grey and the 5′ and 3′ UTR in white. The size (in bp) of the first exon of each transcript is reported. (**a**) Transcripts of *ETV4* curated and listed in NCBI RefSeq database. Three transcripts encode for isoform #1, two transcripts encode for isoform #2, each one of the other 3 transcripts encodes for isoform #3, isoform #4 and isoform #5. Transcripts that encode for the same isoform show different 5' UTR. Isoforms #4 and #5 differ to isoform #1 for the presence of a substitution (amino acid P instead of LA in isoform #4) or a deletion (DGAMG in isoform #5). Transcripts 7 and 8 differ from transcript 1 for some small nucleotide deletions. (**b**) Structure of the hypothetical transcript XM_047435592.1 that should encode for an isoform (X1) of 221 amino acids (24 kDa) due to a start codon in exon 7 restoring the reading frame in spite of a frameshift from skipping of exon 8. (**c**) *ETV4* transcripts assessed in several human cancer (prostate: DU145, PC3, VCap, LNCap, 22RV1; breast: MDA-MB-231 indicated as MDA231, MCF7; leukemia: K562), and normal (prostate RWPE and breast MCF10) cell lines by RT-PCR. The primers, in exon 3 and 12, used to amplify the retro-transcribed *ETV4* cDNA are reported in Supplementary Table. *GAPDH* was used as amplification control. H20: reagent control; M: 100 bp ladder. The sizes of the expected bands are reported on the right.

NCBI Reference Sequences (RefSeq) database^56^ for the human *ETV4* (NC_000017.11 based on GRCh38.p13 Primary Assembly) reports 9 curated transcripts (one listed as “hypothetical”), which encode for 6 putative isoforms (Table 1, Fig. 1a,b).

**Table 1:** List of *ETV4* transcripts reported in NCBI Reference Sequences database and their presence in human cell lines.

Transcript variant	NCBI reference sequence		Putative proteins				Absent in cell line*
	Transcript	Protein	Isoform	aa (*n*)	MW (kDa)	Putative ATG (exon)	
1	NM_001986.4	NP_001977.1	1	484	54	2	RWPE*
2	NM_001079675.5	NP_001073143.1	1	484	54	2	None*
6	NM_001369366.2	NP_001356295.1	1	484	54	2	All*
3	NM_001261437.3	NP_001248366.1	2	445	50	3	22RV1, RWPE
4	NM_001261438.3	NP_001248367.1	2	445	50	3	All*
5	NM_001261439.3	NP_001248368.1	3	207	23	9	None*
7	NM_001369367.2	NP_001356296.1	4	483	54	2	PC3§
8	NM_001369368.2	NP_001356297.1	5	479	53	2	PC3§
Hypothetical	XM_047435592.1	XP_047291548.1	X1	221	25	7	None*

The NCBI Reference Sequences (RefSeq) sequence number and the characteristics of the putative proteins (including the exon in which the start codon is located) are reported for each transcript and protein isoform.

The isoform X1 in which the exon 8 is skipped is reported in the NCBI RefSeq database as predicted protein. aa: amino acids; *n*: number; MW: molecular weight; kDa: kilodalton.

*The investigated cells lines are: PC3, Du145, RWPE, 22RV1 (prostate); MDA-MB-231 (breast), K562 (myeloid leukemia); § only PC3 and Du145 have been tested.

Seven of these NCBI RefSeq curated transcripts correspond to long canonical *ETV4* transcripts (Fig. 1a): (i) transcripts 1, 2 and 6 differ in the 5’ UTR and encode the full-length ETV4 isoform #1 (484 aa); (ii) transcripts 3 and 4 share the 5’UTR with transcripts 2 and 1, respectively, but skipping of exon 2 should lead to a putative 445 aa isoform #2, with a start codon in exon 3; (iii) transcripts 7 and 8 are derivatives of transcript 1 from which they differ for a small in frame deletion of, respectively, 3 (isoform #4) and 15 nucleotides (isoform #5). The short transcript 5 should encode a putative 200 aa isoform #3 due to a start codon in exon 9 (Fig. 1a). Finally, NCBI RefSeq also reports a hypothetical transcript XM_047435592.1 that, in spite of a frameshift due to exon 8 skipping, should encode for an isoform (#X1) of 221 amino acids (24 kDa) due to a predicted start codon in exon 7 (Fig. 1b).

RT-PCR amplification of *ETV4* cDNA from several prostate cell lines using primers in exon 3 and 12 (Supplementary Table 1) has shown the expected 1031 bp amplicon, corresponding to the above long *ETV4* transcripts, and also several shorter amplicons that suggest the presence of additional transcripts (Fig. 1c). Expression of these shorter *ETV4* amplicons has been found also in breast (MDA-MB231) and leukemic (K562) cell lines. In all investigated cell lines the shorter *ETV4* amplicons were always fainter than the 1031 bp ETV4 amplicon. Cell lines in which *ETV4* protein was not detected (VCap, LNCap and MCF7) were not investigated further (data not shown).

**Figure 2:**
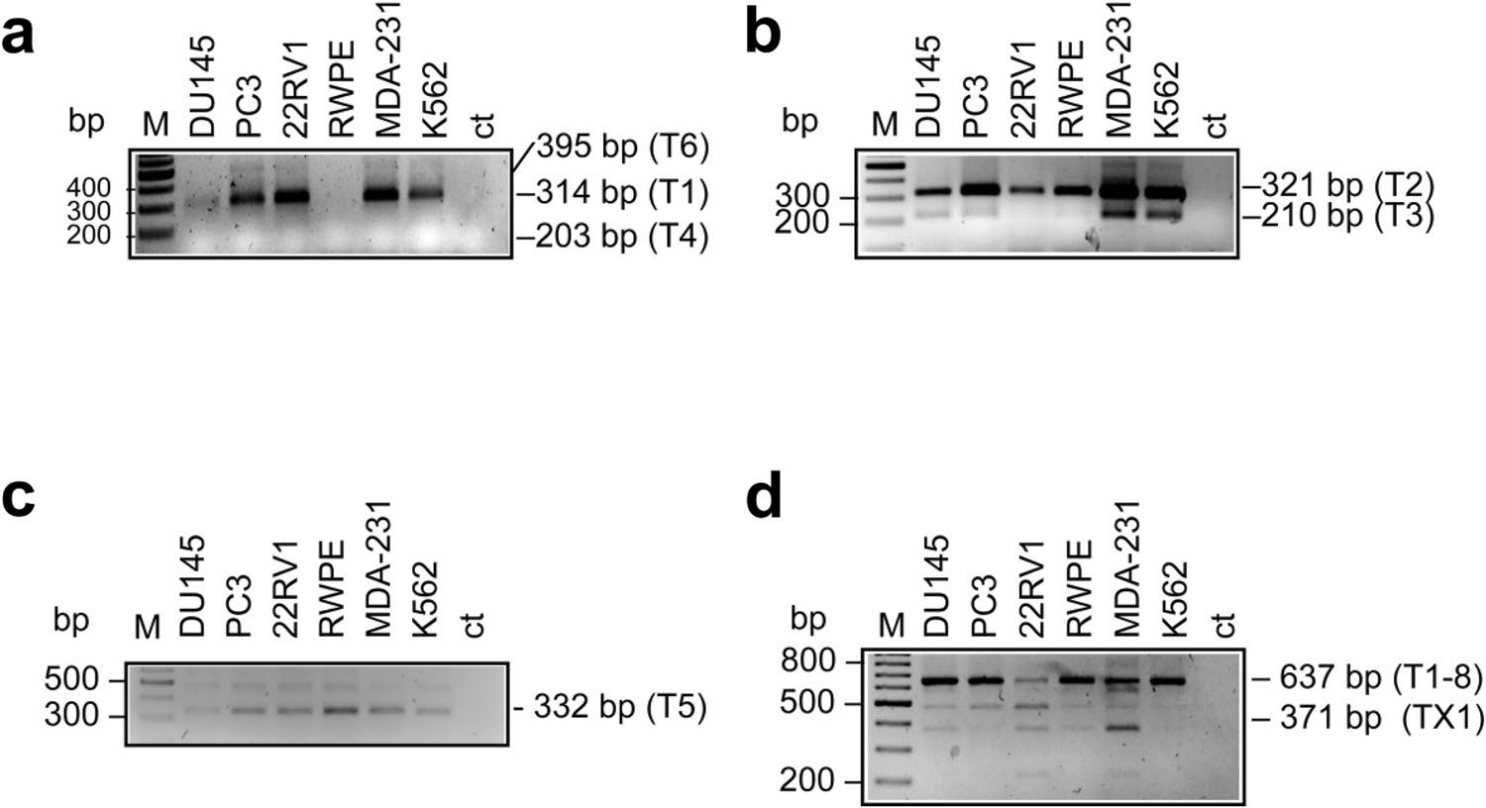
Specific RT-PCR for detection of the *ETV4* transcripts reported in the NCBI RefSeq database in selected cell lines (Du145, PC3, 22RV1, RWPE, MDA-231 and K562). (**a**) RT-PCR to detect Transcript 1 (T1, 314 bp), Transcript 4 (T4, 203 bp) and Transcript 6 (T6, 395 bp). Primers are located in the 5’UTR common to transcripts 1, 4 and 6, and in exon 5. The 314 bp amplicon (Transcript 1) was present in all the analyzed cell lines, except RWPE, while the 203 bp (Transcript 4) and the 395 bp (Transcript 6) amplicons were not detected in any of them. The band corresponding to the 314 bp amplicon may include also Transcripts 7 and 8, which differ from Transcript 1 for the deletion of few nucleotides. (**b**) RT-PCR to detect Transcript 2 (T2, 321 bp) and Transcript 3 (T3, 210 bp). Primers are located in the 5’UTR common to transcripts 2 and 3, and in exon 5. The 321 bp amplicon (Transcript 2) was present in all the analyzed cell lines. The 210 bp amplicon (Transcript 3) was present in all the analyzed cells (faint in RWPE) but in 22RV1. (**c**) Transcript 5 (T5, 332 bp) was detected in all analyzed cells. Primers are located in the 5’UTR of transcripts 5, and in exon 5. (**d**) RT-PCR to detect Transcripts 1 to 8, but 5, (T1-8, 637 bp) and Transcript X1 (371 bp), which were present in all the analyzed cells. Primers are located in exon 6 and in exon 10. The presence of additional amplicons suggests the existence of other alternative transcripts such as *ETV4* ∆7 (475 bp), see Fig. 4. Primer sequences for the specific amplification of each transcript are reported in Supplementary Table 1. Lane M is the 100 bp ladder. Sizes of the expected bands are reported on the right. Ct: reagent control.

The presence of each *ETV4* transcript reported in the NCBI RefSeq database was tested by using specifically designed primers, as reported in Supplementary Table 1. We found that almost all cell lines express the transcripts 1 (Fig. 2a), 2, 3 (Fig. 2b) and the short transcript 5 (Fig. 2c), with the notable absence of transcript 3 in 22RV1 (Fig. 2b) and of transcripts 1 and 3 in RWPE (Fig. 2a,b). We have not been able to detect transcript 4 and 6 in any of the cell lines (Fig. 2a). Finally, in all the investigated cell lines we have found an amplicon corresponding to the hypothetical XM_047435592.1 transcript (isoform #X1) that from now on will be named X1 (Fig. 2d).

**Figure 3:**
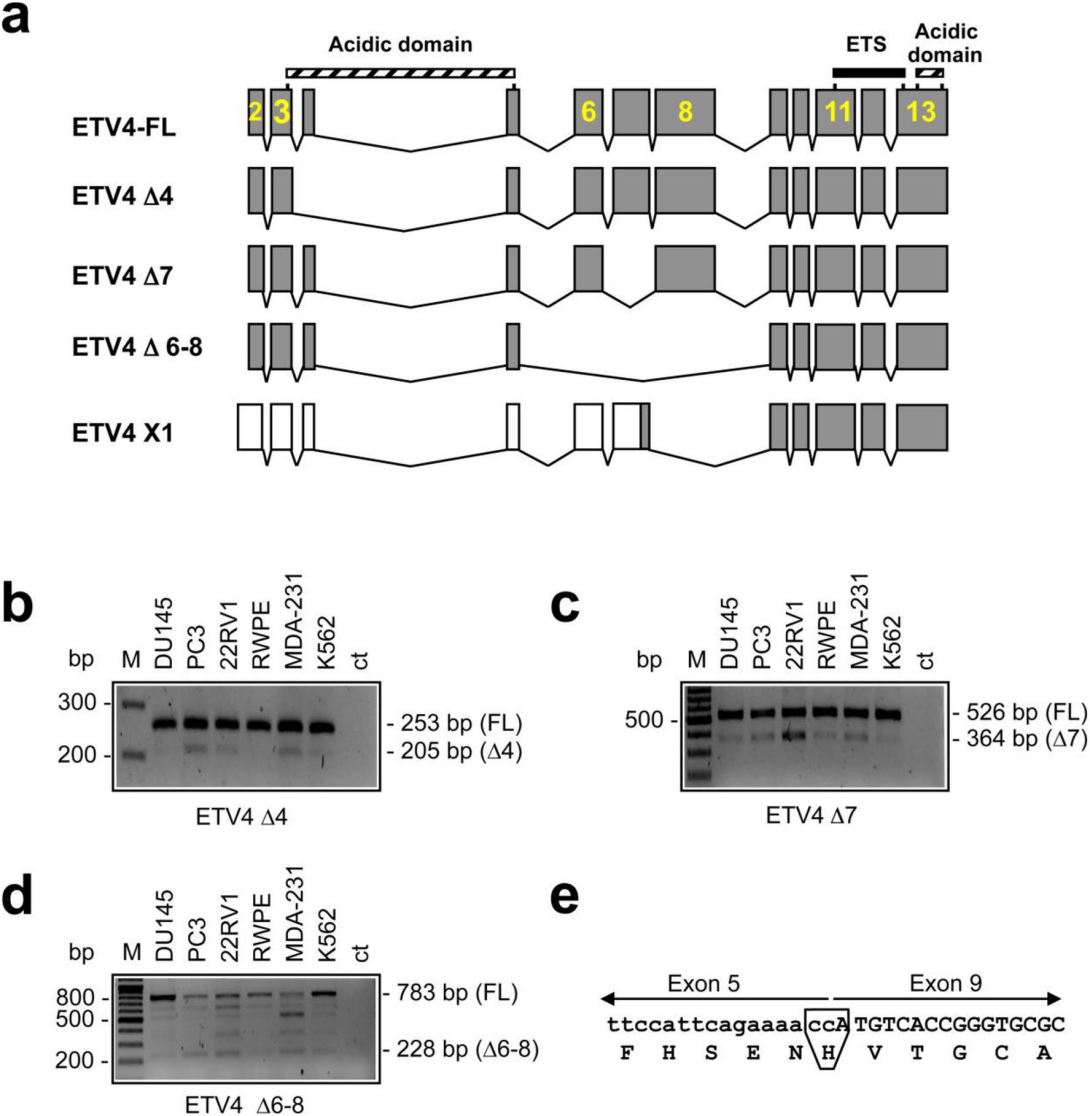
Detection of the novel *ETV4* alternative transcripts in selected cell lines (Du145, PC3, 22RV1, RWPE, MDA-231 and K562). (**a**) Schematic representation of the potentially productive splice transcripts of *ETV4* found by cloning and sequencing in PC3 and Du145 cell lines. The exon structure is shown with coding exon sequences in grey and 5’ UTRs in white. The position of functional features is shown at the top: DNA-binding ETS motif (black bar); Acidic domain (hatched bars). The structure of the full-length *ETV4* (*ETV4* FL) and of the short X1 transcript (*ETV4* X1) are reported for comparison. *ETV4* ∆4, deletion of exon 4; *ETV4* ∆7, deletion of exon 7; *ETV4* ∆6–8, deletion of exons 6, 7 and 8. (**b-d**) Specific RT-PCR for detection of the novel alternative transcripts of *ETV4* in cell lines. Primer sequences for the specific amplification of each alternative transcript are reported in Supplementary Table 1. Lane M is the 100 bp ladder. Sizes of the expected bands are reported on the right. In each panel the larger band is from the long *ETV4* transcripts (*ETV4*-FL), the smaller from *ETV4* ∆4 (**b**), from *ETV4* ∆7 (**c**) and from *ETV4* ∆6–8 (**d**). In panel (**d**) are present additional bands including the 621 bp (*ETV4* ∆7) and the 517 bp (*ETV4* X1) amplicons. (**e**) Nucleotides present at junction in the *ETV4* ∆6–8 variant. The amino acids present at the junction are also reported. In the box is shown the amino acid substitution presents in the variant in respect to the full-length *ETV4*. Exon numbering is according to the *ETV4* genomic sequence NC_000017.11 based on the GRCh38.p13 Primary Assembly.

Thus, the cell lines we have analyzed express both the short alternative transcripts reported in NCBI RefSeq database (transcript 5 and X1). However, these two short transcripts do not account for the number of short *ETV4* amplicons we have found (Figs. 1c, 2d), suggesting the expression of additional novel *ETV4* transcripts. Identification and characterization of these potentially novel alternative *ETV4* transcripts have been performed by cloning and sequencing the *ETV4* RT-PCR products from PC3 and Du145 cells (primers in exon 3 and 13: Supplementary Table 1). The cloning and sequencing approach confirmed the results obtained with the specific RT-PCR (see above), including the presence of the hypothetical XM_047435592.1 transcript (isoform #X1), and revealed the presence of transcripts 7 and 8 in Du145 but not in PC3 cells (Table 1). Furthermore, this approach revealed also the presence of several additional alternative transcripts (Table 2), few of which having the potential to be transcribed into a protein due to *in frame* exon skipping: a transcript without exon 4 (*ETV4* ∆4), one without exon 7 (*ETV4* ∆7) and one without exons 6, 7 and 8 (*ETV4* ∆6–8) (Fig. 3a). The presence of these three potentially productive transcripts (*ETV4* ∆4, *ETV4* ∆7 and *ETV4* ∆6–8) has been tested by specific RT-PCR (Fig. 3b–d). The transcripts *ETV4* ∆4, *ETV4* ∆7 and *ETV4* ∆6–8 should encode for putative ETV4 proteins of 468 aa (52 kDa), 430 aa (48 kDa) and 299 aa (33 kDa), respectively. In addition, ETV4 ∆6–8 should present the substitution of an aspartic acid with a histidine due to the junction between exons 5 and 9 (Fig. 3e). It is noteworthy that none of these potentially productive alternative *ETV4* transcripts, except *ETV4* ∆7 (ENST00000545089 in the ENSEMBL database), have been described so far.

**Table 2:** List of the additional transcripts found in PC3 and Du145 cell lines by cloning and sequencing.

Transcript sequence features	Putative protein aa, *n* (kDa)	Cell lines
CAG in frame insertion between exons 2 and 3	485 (54)	PC3
Exon 4 skipping	468 (52)	PC3
Exon 7 skipping *	430 (48)	DU145, PC3
Exons 6, 7 and 8 skipping	299 (34)	DU145, PC3,
ATCTCTTCCAG deletion at 5’ end of exon 3	frameshift	PC3
Exons 10 and 11 skipping	frameshift	DU145, PC3

*This transcript was reported as ENST00000545089 in ENSEMBL database. aa, *n*: number of amino acids; kDa: kilodalton.

Finally, in an exploratory experiment we have investigated the relative expression of these alternative *ETV4* transcripts within the total *ETV4* mRNA by quantitative RT-PCR; this very rough quantification showed that alternative transcripts accounted for 23% of total *ETV4* mRNA in PC3 cells and up to 74% in 22RV1 cells (Supplementary Fig. 1). In all prostate cell lines ∆7 was the most expressed among the alternative transcripts, followed by ∆4 and X1, except for the 22RV1 cells, which express X1 at levels similar to ∆7. This expression pattern was also observed in the leukemic K562 cells, whereas the pattern in breast cancer MDA cells was more similar to that of 22RV1 (Supplementary Fig. 1). Due to the difficulty in obtaining reliable relative quantification of all ETV4 splice variants by quantitative RT-PCR, we resorted to long-read sequencing analysis using the Oxford Nanopore platform.

### Explorative analysis of alternative ETV4 transcripts in cell lines by nanopore sequencing

In order to investigate the presence and the distribution of alternative *ETV4* transcripts in an unbiased manner we have amplified the *ETV4* cDNA using a primer in exon 3 and a common external one, followed by sequencing with the Oxford Nanopore platform. Nanopore sequencing has confirmed that the transcripts identified by cloning represent valid *ETV4* transcripts (∆4, ∆7, ∆6–8 and X1) and has also revealed several other alternative transcripts, which possibly could encode for a protein product (Table 3). It is interesting that three of these additional transcripts combine exon 4 skipping with each one of the transcripts identified by cloning. Among alternative ETV4 transcripts identified by Nanopore sequencing, the one with exon 9 skipping was present at a frequency of around 1% in PC3 and RWPE prostate cell line as well as in breast cancer and leukemic cell lines; whereas, in most of the cell lines, all other transcripts have frequency below 0.5%. It is noteworthy that the non-productive ∆7–8 transcript is relatively abundant in all prostate cell lines besides PC3.

**Table 3:** Relative abundance of *ETV4* variant transcripts by NANOPORE sequencing.

Transcripts	aa (*n*)	MW (Da)	PC3	DU145^#^	22RV1	RWPE	MDA	K562
Canonical ETV4*	484 or 445*	53,939 or 49,660*	76.1	23.1	14.72	61.45	66.22	85.63
∆4	468	51,978	11.49	3.23	1.91	4.14	7.13	4.67
∆7	430	48,152	2.91	7.73	14.45	3.18	3.57	1.98
∆9	459	51,470	1.41	*0.32*	*0.24*	1.13	1.19	1.38
∆8(X1)	221	24,716	1.36	25.1	15.06	5.63	11.68	0.84
^§^∆4;∆7	414	46,191	0.63	0.5	1.28	*0.12*	*0.21*	*0.12*
∆6–8	299	33,600	*0.39*	15.6	26.35	12.42	0.98	0.6
^§^∆4;∆8(X1like)	221	24,716	*0.15*	1.82	0.82	*0.32*	2.17	*0.12*
^§^∆4;∆6–8	283	31,639	*0.1*	1.96	2.94	1.45	*0.14*	–
∆5–9	256	29,065	*0.05*	4.82	*0.36*	0.72	–	–
∆5–8	281	31,535	*0.05*	1.5	0.64	*0.48*	*0.07*	–
∆6–9	274	31,207	*0.05*	1.32	0.85	0.6	–	–
∆7–8	Frameshift		*0.24*	4.23	11.6	2.49	0.91	*0.12*
^§^∆4;∆7–8	Frameshift		*0.05*	*0.41*	1.31	*0.08*	*0.07*	*–*
∆6;∆8	Frameshift		–	1.68	0.88	*0.24*	*0.35*	*0.06*
Others			5.02	6.68	6.59	5.55	5.31	4.48

Only transcripts found with a frequency ≥ 1% in at least one cell line are shown. The frequency of transcripts under 1% in a specific cell line is reported in italics. A dash (–) indicates that the transcript has not been found.

*This transcript includes the canonical ETV4 transcripts with the start codon either in exon 2 (484 aa) or in exon 3 (445 aa).

^§^Transcripts that combine exon 4 skipping with exons skipping found in other variant transcripts.

^#^*ETV4* template of Du145 cell line was obtained with 35 cycles of PCR amplification. As such these sequencing data are qualitatively but not quantitatively reliable.

Others: the sum of the frequency of transcripts not reported in this table and with a frequency < 1.0%.

*aa*: amino acids; *n*: number; *MW*: molecular weight; *Da*: dalton; *MDA*:MDA-MB231.

Nanopore sequencing allowed quantification of *ETV4* transcripts (Table 3): among prostate cell lines canonical *ETV4* were the most abundant transcripts in PC3 (76.1%) and RWPE (61.5%) and also in breast cancer (66.2%) and leukemic (85.2%) cell lines. Whereas in 22RV1, the most expressed transcript was the ∆6–8 (25.1%). Overall, these results were similar to the quantification obtained by quantitative RT-PCR (Supplementary Fig. 1).

### Alternative ETV4 transcripts in patients with prostate cancer

In order to assess whether these novel alternative *ETV4* transcripts were also present in prostate cancer tissues, we retrieved from the Cancer Genome Atlas (TCGA, https://portal.gdc.cancer.gov/) the data on tumor prostate tissues from prostate cancer patients whose tumor tissue expressed *ETV4*. This analysis confirmed that most of the novel alternative *ETV4* transcripts found in cell lines were also present in the tumor tissues of these patients (Table 4): ∆7 and ∆8(X1) transcripts were found in most of the patients (82 and 68%, respectively) and in 64% of patients were present both; also ∆4 and ∆6–8 transcripts were relatively frequent (25 and 32%, respectively). These four transcripts were present all together in 21% of patients. None of these transcripts was found in the tumor tissue of 18% of patients and in the normal peritumoral tissues of 60% of patients. In comparison, alternative transcripts were found in normal peritumoral tissues in a relatively small fraction (40%) of PC patients and only at low expression levels (Table 4): in any event, also in the “normal” peritumoral tissue the ∆7 and ∆8(X1) transcripts were the most frequent (23 and 17%, respectively).

**Table 4:** *ETV4* transcripts with exon skipping found in tumor and peritumoral samples of prostate carcinoma patients from TCGA dataset.

Exon junction*	Putative transcript*	Putative protein	Tumor tissue from PC patients (%)	Peritumoral tissue from PC patients (%)
3–5	∆4	yes	25.0	1.9
6–8	∆7	Yes	82.1	23.1
5–9	∆6–8	Yes	32.1	–
7–9	∆8 (X1)	Yes	67.9	17.3
4–6	∆5	Yes	7.1	–
5–7	∆6	Yes	25.0	–
6–9	∆7–8	Frameshift	35.7	1.9
8–10	∆9	Yes	7.1	–
9–11	∆10	Yes	17.9	–
11–13	∆12	Yes	7.1	–

*Exon junctions (and their associated putative transcripts) found in the tumor tissue of at least 2 patients with a total of more than 20 reads.

Of note the number of reads of these selected exon junctions in peritumoral tissues was ≤ 18 (range 1–18).

### Characterization of the isoforms translated from the alternative ETV4 transcripts

**Figure 4:**
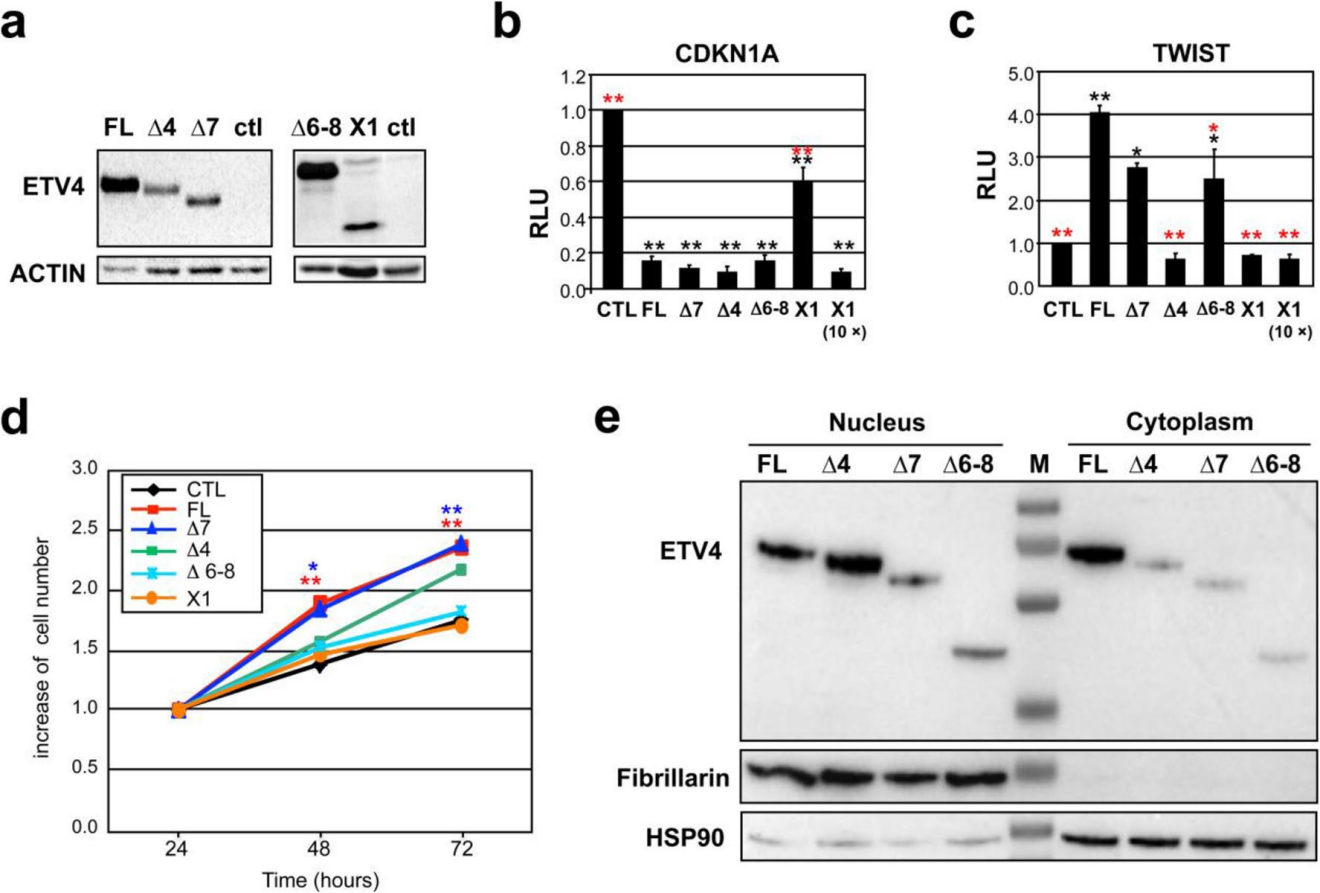
Functional characterization of proteins translated from the alternative *ETV4* transcripts. (**a**) Western blot analysis of ETV4 isoforms expressed from 4 specific expression vectors: ETV4-∆4, ETV4-∆7, ETV4-∆6–8, and ETV4-X1. FL, cells transfected with a vector expressing the full-length *ETV4* (ETV4-FL). CTL, cells transfected with the empty vector. (**b-c**) *ETV4* splice variants transcriptional activity measured by luciferase reporter assays. The vectors encoding each one of *ETV4* splice variants or ETV4-FL have been transfected in RWPE cells along with a vector containing part of *CDKN1A* (**b**) or *TWIST1* (**c**) promoter upstream firefly luciferase. Relative luciferase units (R.L.U.) is the firefly/renilla ratio of each variant compared to that obtained by control (CTL, cells transfected with the empty vector). The data represent mean ± sem from at least three independent experiments performed in triplicate: *P < 0.05, **P < 0.01 (black asterisks: comparison with CTL; red asterisks: comparison with *ETV4* FL). (**d**) Cell proliferation assessed by MTT assay in RWPE cells expressing the ETV4 isoforms. The values are expressed as increase of the initial cell number. Each value represents the average of 3 independent experiments. Cells transfected with any of ETV4-FL, ETV4 ∆7 and ETV4 ∆4 showed an increase of the proliferation rate, although this increase was statistically significant only for ETV4-FL and ETV4 ∆7 *P < 0.05, **P < 0.01. (**e**) Expression of the ETV4 isoforms in the nucleus (left) and in the cytoplasm (right) of RWPE cells transiently transfected with the indicated ETV4 isoforms (∆4, ∆7 and ∆6–8) compared with the ETV4-FL (FL). Fibrillarin and HSP90 have been used as loading control for nucleus and cytoplasm, respectively. M: molecular weight marker.

In order to analyze the biological characteristics of the putative isoforms translated from the novel *ETV4* transcripts (∆4, ∆6–8, ∆7, and X1), we have cloned each of them in four expression vectors. These vectors, upon transient transfection, were able to express proteins of the expected size (Fig. 4a). Transcription ability, proliferation ability and subcellular localization of these isoforms have been compared with the full-length ETV4 (ETV4-FL)^34^ in transfected cells (Fig. 4b–e)**.**

#### Transcription ability of ETV4 isoforms

We have investigated the transcriptional activity of these ETV4 isoforms using a dual luciferase reporter assay. We co-transfected RWPE normal prostate cells with the expression vector containing the coding sequence of each one of the above *ETV4* transcripts alongside with one containing, upstream to the firefly gene, a fragment of the promoter of two known targets of ETV4. Specifically, we used the promoters of *TWIST1*^57^ and *CDKN1A* (P21)^35^ genes, which are, respectively, positively and negatively regulated by ETV4. We compared the relative luciferase units (R.L.U.) of each ETV4 isoform with ETV4-FL (Fig. 4b, c). All these ETV4 isoforms, except X1, were able to reduce luciferase expression driven by a region of *CDKN1A* (P21) promoter with the same efficiency as the full-length ETV4 (ETV4-FL) (Fig. 4b). The weaker inhibition by X1 could be explained by the reduced amount of protein yielded by the expression vector: indeed, its inhibition ability was fully restored by increasing the amount of transfected vector (Fig. 4b). In contrast, ETV4-∆7 and ETV4-∆6–8 but not ETV4-∆4 and ETV4-X1 were able to increase luciferase expression driven by a region of *TWIST1* promoter (Fig. 4c). Transfection with increased amounts of ETV4-X1 plasmid did not modify the effect of X1 on the *TWIST1* promoter.

#### Proliferation induced by the novel ETV4 isoforms

We compared the ability of these ETV4 isoforms to increase the proliferation rate of RWPE cells. Transfection with either ETV4-∆7 or ETV4-∆4 was able to increase the proliferation rate of RWPE cells at levels similar to those induced by ETV4-FL (although only ETV4-FL and ETV4-∆7 reached a significant difference compared to control–RWPE cells transfected with empty vector). On the contrary, ETV4-∆6–8 and ETV4-X1 were unable to increase RWPE proliferation rate (Fig. 4d).

#### Subcellular localization of the novel ETV4 isoforms

Analysis of the subcellular localization of these isoforms in the RWPE cell line has shown that they were located mainly in the nucleus (Fig. 4e) compared to ETV4-FL, which was more homogenously distributed in both nuclear and cytoplasmatic compartments. It is interesting that a similar pattern has been observed also in the MCF7 breast cancer cell line (Supplementary Fig. 2).

## Discussion

The impairment and the dysregulation of the splicing machinery have been recognized as one of the key features that characterize cancer cells. In fact, the presence of alternative mRNA splicing transcripts is quite common in cancer and can result either from mutations in splicing regulatory elements or from changes in the regulatory splicing machinery^58,59^. Several genes exhibit abnormal splicing pattern in cancer and, often, specific patterns may characterize particular types of cancer. Similar findings have been reported also for ETS transcription factors that play a pathogenic role in several kind of cancers^10^. In fact, alternative splicing, modifying the arrangement of ETS regulatory domains, may generate variants of an ETS transcription factor with different functions and different oncogenic potential^39,51,54,60^ that, eventually, can influence prognosis^53^.

ETV4 is an ETS transcription factor that plays an important oncogenic role in several tissues, especially in prostate and breast. Nine different *ETV4* transcripts have been previously described, seven of which generate very similar canonical isoforms because of an alternative usage of 5′UTR (Fig. 1a,b; Table 1). However, compared with other ETS transcriptional factors, there is no data on the usage of alternative *ETV4* transcripts in normal and cancer cells. Here, for the first time, the expression pattern of *ETV4* alternatively spliced transcripts has been systematically analyzed.

Systematic analysis of the known *ETV4* transcripts reported in RefSeq revealed that the pattern of expression of the canonical transcripts was variable in different prostate cell lines, whereas all cell lines expressed both the short transcripts (transcript 5 and X1). However, these two short transcripts do not explain the number of short fragments obtained from the amplification of the entire *ETV4* mRNA (Fig. 1c). In fact, unbiased cloning and sequencing of the *ETV4* RT-PCR products confirmed the presence of the known transcripts and further revealed that prostate cell lines express several novel alternative transcripts. Most of these transcripts could be transcribed into an ETV4 protein as a result of an in frame exon skipping (Table 2): exon 4 for ETV4 ∆4, exon 7 for ETV4 ∆7 and exons 6, 7 and 8 for ETV4 ∆6–8 (Fig. 3a).

In order to confirm these findings and to identify other possible alternative *ETV4* transcripts we used high-throughput sequencing to perform an unbiased survey. Typically, with second-generation sequencing, untargeted transcript identification is performed through assembly of short reads spanning the gene of interest and extrapolation of the structure of the transcript based on splice junctions and read frequencies. These approaches may introduce bias and artifacts that could impede the determination of the real structure of the transcripts^61^. To avoid such possible bias and artifacts, we have used the Oxford Nanopore Technology platform, which is able to sequence long fragment of genetic material allowing precise characterization of the structure of the transcripts and quantification of their expression^62,63^.

Nanopore sequencing has confirmed that the transcripts identified by cloning represent valid *ETV4* transcripts (∆4, ∆7, ∆6–8 and X1) and has revealed additional alternative transcripts that include both potentially productive (*ETV4* ∆9 and those combining exon 4 skipping with each one of ∆7, ∆6–8 and X1) and non-productive (*ETV4* ∆7–8 and ∆6;∆8) transcripts (Table 3). Analysis of *ETV4* transcripts in tumor and peritumoral samples of prostate carcinoma patients from TCGA dataset revealed the presence of exon-to-exon junctions compatible with the *ETV4* transcripts identified by cloning and nanopore sequencing (Table 4): this provides an additional, although indirect, evidence that they represent valid *ETV4* transcripts. In addition, Nanopore sequencing has allowed also a more precise relative quantification of *ETV4* transcripts (Table 3) with the respect to quantitative RT-PCR. Canonical *ETV4* transcripts were more abundant than all other alternative transcripts in prostate cell lines PC3 (76.1%) and RWPE (61.5%) but not in DU145 (23.1%) and 22RV1 (14.7%). The patterns of expression of the potentially productive alternative transcripts varied in the investigated prostate cell lines: ∆4 was the most expressed in PC3 whereas X1 and ∆6–8 were the most frequent in the other cells (DU145, RWPE and in 22RV1). It is noteworthy that canonical, ∆7, X1, and ∆6–8 transcripts were expressed at similar levels in 22RV1 (∆6–8 was the most frequent) and that in this cell line also the non-productive ∆7–8 transcript, barely found in the other prostate cell lines, was relatively abundant (11.6%). For comparison, canonical *ETV4* transcripts are the most abundant also in breast cancer (66.2%) and leukemic (85.2%) cell lines.

Various distinct molecular mechanisms may cause alternative splicing: activation of alternative 5′ and 3′ splice sites, intron retention, exon skipping and alternative exon usage. Most of previously known *ETV4* transcripts encode for slightly different isoforms differing in the start codons because of the alternative usage of either exon 2 or exon 3 (Table 1). On the other hand, almost all the novel alternative *ETV4* transcripts found in prostate cell lines and in human prostate tumors derive from exon skipping mechanism (Tables 2, 3 and 4). All the potentially productive alternative *ETV4* transcripts retain the ETS domain, encoded by exons 11, 12 and 13: thus, their encoded isoforms should retain the ability to drive transcription. Nevertheless, these splice variants could potentially have different biological roles compared to the canonical isoforms thanks to loss of inhibitory or activating sequences in the skipped exons. It is also possible that exon skipping could affect ETV4 mRNA/protein stability or its ability to localize in the nucleus where ETV4 exerts the transcriptional activity.

The biological properties of the putative proteins encoded by the alternative transcripts, which are more frequent in prostate cell lines and in tumor tissues (*ETV4* ∆4, ∆6–8, ∆7, and X1) have been investigated in vitro. Transfection experiments in prostate cell lines showed that the ability to increase proliferation rate was retained only by ETV4 ∆7 and, to a lesser extent, by ETV4 ∆4 (Fig. 4d), in spite of the fact that all splice variants were preferentially located into the nucleus (Fig. 4e). We have previously shown that ETV4 is able to promote prostate cell proliferation also through p21 downregulation mediated by direct binding of ETV4 to the *CDKN1A* (p21) promoter^35^. Therefore, it is somehow surprising that, despite their very different ability to promote cell proliferation, all these splice variants were able to exert a negative regulation of *CDKN1A* (P21) promoter with efficiency apparently comparable to the canonical ETV4 (Fig. 4b). This confirms that the ability of ETV4 to promote proliferation is only partially due to direct downregulation of the *CDKN1A* (p21) promoter^35^.

ETV4, analogously to the other member of the ETS family, interacts with various complexes of transcriptional co-factors and contains multiple regulatory domains that modify its translational capability and allows a tight regulation of the target genes. The N-terminal inhibitory domain (NID), spanning ETV4 exons 7 and 8, is one of the intramolecular autoinhibitory modules that regulates ETV4 activity: in fact, NID represses ETV4 DNA binding via transient interactions with the DNA-recognition helix of the ETS domain, interaction that is released by the acetylation of Lysine (Lys226 or Lys260)^64^. However, lack of a large portion of NID did not result in a significant increase of the effects on ETV4 target promoters. In fact, the *ETV4* variants missing exons 7 and 8 (∆6–8 and ∆7) did not show, in comparison with canonical variants, any increased ability in repressing the *CDKN1A* (P21) promoter or in promoting the *TWIST1* promoter (Fig. 4b,c). In addition, despite a discrete effect on the *CDKN1A* promoter repression, ETV4 Δ6-8 was unable to promote cell proliferation. This suggests that exons 7 and 8 include, together with the autoinhibitory NID, also additional structures with diverse and likely opposite functions.

Exon 4 encodes for a large part of the amino-terminal transactivation domain (TAD) responsible for the interaction of ETV4 with MED25, a subunit of the evolutionary conserved multi-subunit RNA polymerase II transcriptional regulator named Mediator^65^. It is intriguing that the two splice variants lacking exon 4, ETV4 ∆4 and ETV4 X1, were unable to exert the positive regulation of the *TWIST1* promoter (Fig. 4c). Thus, it is possible that absence of exon 4 (ETV4 ∆4, ETV4 X1) prevents ETV4 binding to MED25 and –consequently– to Mediator, thus hindering transcriptional activation of the *TWIST1* promoter. Moreover, all the investigated splice variants, irrespective of exon 4 status, were able to downregulate the *CDKN1A* (P21) promoter suggesting that TAD-Mediator interaction is not necessary for the negative regulation of this and, possibly, other promoters, e.g. *ERBB2 and* collagenase-1, usually negatively regulated by ETV4^66,67^. This phenotypic characterization of the alternative ETV4 variants has revealed subtle differences in their biological features that could provide insight in the functional organization of ETV4. However, a deeper functional characterization is still needed, especially to evaluate whether the interaction between canonical and alternative ETV4 variants could affect their function similarly to what as has been shown for other ETS proteins^68^.

None of these splice variants have completely lost their transcriptional ability, yet our partial functional characterization does not seem to support the notion that some ETV4 alternatively spliced variants have higher neoplastic potential with respect to the canonical variants. Nevertheless, at least one alternative *ETV4* transcript is expressed in the tumor tissues of most of prostate cancer patients (82%) and one in four of these patients expressed at least 4 different alternative transcripts; at variance, alternative *ETV4* transcripts were found expressed, usually at very low levels, in the peritumoral tissues of only 40% of patients. Thus, the abundance of alternative *ETV4* transcripts seems specific of prostate tumors.

In conclusion, we have identified and characterized a previously unknown set of *ETV4* alternatively spliced transcripts, most of which are expected to encode ETV4 protein isoforms. These transcripts are much more abundant in human prostate tumors than in normal tissues. This pattern of expression with such abundance of alternative *ETV4* transcripts in prostate tumors is likely to be secondary to the deranged splicing machinery often present in tumors. However, although none of the alternative splice variants appear to have acquired key neoplastic abilities, it is possible that molecular interactions between canonical and alternative variants contribute to influence the neoplastic features of prostate cancer. Further investigations are needed to completely define the role, if any, of the alternative ETV4 variants in defining the features and the development trajectories of prostate cancer.

## Materials and methods

### Cell culture

Du145, PC3, LnCap, MCF7, MDA-MB231, K562 (IST “Cell Bank and Cell Factory”, Genoa, Italy), V-Cap, 22RV1, RWPE, MCF10 (American Type Culture Collection) human cell lines were cultured according to cell-bank instructions. Du145, LnCap, 22RV1 and K562 cell lines were maintained in RPMI-1640 medium; MDA-MB231, MCF7 and VCap cell lines were maintained in DMEM high glucose medium; PC3 cell line were maintained in HamF12 medium; all these media were supplemented with 10% fetal bovine serum, 4 mM of L-glutamine, 100 U/ml penicillin, 100 µg/ml streptomycin. RWPE cell line was maintained in serum-free KER medium supplemented with Epidermal Growth Factor and Bovine Pituitary Extract (Gibco, Carlsband, CA, USA). MCF10 cell line was maintained in DMEM medium, supplemented with 20% fetal bovine serum, 25 U insulin, 0.5 μg/mL hydrocortisone and 5 nM EGF (Gibco). RWPE and MCF10A media were supplemented with 4 mM of L-glutamine, 100 U/ml penicillin, 100 µg/ml streptomycin.

### Reverse transcription polymerase chain reaction (RT-PCR)

Total RNA was extracted with RNeasy Mini Kit (Qiagen, Germantown, MD, USA) and reverse transcribed using the Gene Amp RNA PCR Kit (Life Technologies-Thermo Fisher Scientific, Monza, Italy). Briefly, 1 µg of total RNA, in a reaction mixture (total volume 20 µl) containing 2.5U of reverse transcriptase with its buffer, 2.5 uM of random-examer primers and 100 µM of each deoxynucleoside triphosphates (dNTP) (Life Technologies), was reverse transcribed at 42 °C for 40 min; the enzyme was inactivated at 99 °C for 5 min, and the samples were immediately chilled at 4 °C.

The cDNA was used as template to amplify the *ETV4* transcripts with the primers listed in Supplementary Table 1. Briefly, 1 µl of the above template was amplified in 50 µl reaction mixture containing 1.25 U of Taq Polimerase with its buffer and 2 mM MgCl_2_ (Life Technologies-Thermo Scientific), 100 µM of each dNTP, 0.4 µM of each primers. PCR amplification was performed for 35 cycles (94 °C for 20 s, 57 °C for 20 s, and 72 °C 45 s) on MyCycler (Bio-Rad Hercules, CA, USA).

### Identification of ETV4 splice variants and construction of variant expression vectors

In order to identify possible *ETV4* splice variants, *ETV4* cDNAs from the RNA from PC3 and DU145 cell lines were amplified (see above) by primers in exon 3 and 13 (Fig. 1a) and the products were cloned using the TOPO TI cloning Kit into the pCRII cloning vector (Life Technologies) according to the manufacturer's protocol. The cloned inserts were characterized by Sanger sequencing. The presence of the splice variants identified by this approach was tested in the various cell lines by RT-PCR using the primers specific for each variant, which are listed in Supplementary Table 1.

Some of the identified novel variants (*ETV4* ∆4, ∆7, ∆6-7-8, and X1: see “Results” for details) were cloned in a previously described expression vector encoding the full-length ETV4 (ETV4-FL) corresponding to the NCBI Transcript 1^34^**.** Specifically, the BtrI-FspAI fragment contained in the *ETV4*-FL vector was replaced with the BtrI-FspAI fragment from the pCRII plasmids containing the splice variants. Each vector has been sequenced. Sequence and exon numbering is according to the NC_000017.11 sequence based on the GRCh38.p13 Primary Assembly.

### Reverse transcription quantitative PCR (RT-qPCR)

ETV4 transcripts were quantified by RT-qPCR performed on CFX96 thermocycler (Bio-Rad Hercules, CA, USA). Briefly, 3 µl of a 1:30 dilution of the cDNA template, produced as described above, was used in a qPCR reaction mixture (total volume, 20 µl) containing the SsoFast EvaGreen Supermix (Bio-Rad) and 0.3 uM of each primer (see below and supplementary Table 1). Thermal conditions: 98 °C for 30 s followed by 45 cycles (98 °C for 6 s, 60 °C for 10 s). Relative expression levels of the target genes were calculated by the 2^-ΔΔCt^ method using the glyceraldehyde phosphate dehydrogenase (*GAPDH*) as reference gene. Each RT-qPCR has been performed 3 times in triplicate.

To quantify ETV4 splice variants we designed, for each variant, a primer recognizing only the specific variant paired with another appropriate primer. Primer specificity is due to its complementarity to the sequence of the junction derived from the alternative splicing. Primer pairs for *ETV4* ∆4, ∆7, ∆6-7-8, and X1 splice variants are listed in Supplementary Table 1.

In order to roughly quantify total *ETV4* transcripts we designed a pair of primers that amplifies an ETV4 amplicon from exon 11 to exon 12, which encompasses the ETS domain and is present in all variants: thus, it may serves as a proxy for total *ETV4* transcripts.

To estimate relative proportions of each splice variant in the respect of the total *ETV4* transcripts in a cell line, the relative expression level of each variant was normalized against that of the total *ETV4* transcripts (see above). However, since the amplification efficiency of each amplicon has not been demonstrated this technique provides only a very rough estimate of the proportion of the splice variant in each cell line. After this exploratory experiment a proper quantification of the expression of ETV4 splice variants was performed by Nanopore sequencing (see below).

### Cell transfection

Transient transfection was performed on 70% confluent cells using 5 μg of expression vector (ETV4-FL, ETV4 ∆4, ∆7, ∆6-7-8 and X1) and 12 μl of X-tremeGENE HP DNA transfection reagent (Merck, Darmstadt, Germany) according to manufacturer instructions. Cell lysates were prepared and analyzed 48 h after transfection.

### Identification and quantification of ETV4 transcripts by Nanopore Sequencing

Total RNA (1–3 µg) was extracted and retro-transcribed using a reverse primer containing a sequence complementary to an *ETV4* sequence (exon 13: 5′-AGTGGGACAAAGGGACTGTG-3’), a specific barcode for each cell line followed by a common shared sequence (5’-GACCACGCGTATCGATGTCGAC-3’). The reaction mixture (total volume 20 µl) contained 100 U of Maxima H minus Reverse transcriptase with its buffer (Life Technologies), 20 U of RNAse inhibitor, 500 µM of each dNTP (Life Technologies) and 20 pmol of the above primer. *ETV4* cDNA was amplified using a forward primer in exon 3 (5′-GCCGCCCCTCGACTCTGAA-3′) and a reverse primer containing only the common shared sequence (see above). The reaction mixture (total volume 50 µl) contained 1 U of the high fidelity thermo-stable KOD Hot-start DNA polymerase with its buffer and 1.5 mM of MgSO_4_ solution (Merck), 200 µM of each dNTP, 0.3 µM of each primer. To maintain a linear proportion of each transcript, *ETV4* cDNA was subjected to 20 amplification cycles. *ETV4* cDNA from Du145 cell line was subjected to 35 amplification cycles, allowing only a qualitative analysis of *ETV4* transcripts.

Barcoded PCR products were purified using 1.8 X Agencourt AMPure XP beads (Agentcourt). Purified PCR products were quantified using Qubit High Sensitivity Kit (Thermo Fisher, Waltham, MA, USA). Based on their concentrations, equimolar concentration of the PCR products were pooled together. Libraries were prepared from these pooled PCR products using the SQK-LSK109 kit (Oxford Nanopore Technologies), according to the manufacturer protocol. The libraries were run on Flongle flow cells (v.9.4.1 pore), using the MK1B sequencer on the MinKNOW software (v.19.2.2) (Oxford Nanopore Technologies). Base calling was performed using GUPPY (v.3.0.3) to generate FASTQ files from the FAST5 files. Reads were then aligned to the reference genome and analyzed using minimap2 (v.2.17)^69^. For IGV visualization, BAM files were sorted according to mapped chromosomal location using sort from SAMtools^70^ to visualize the reads mapped on to the reference genome^71^**.** Moreover, sequence reads were aligned to each individual barcode and exon/intron on the *ETV4* gene using LAST (version 1045)^72,73^:

lastdb -uNEAR -cR01 target target.fasta.

last-train -Q1 target reads.fastq > target.par.

lastal -P4 -Q1 -p target.par target reads.fastq | last-split -m1 > myaln.maf.

The alignment files were then used to demultiplex the reads and to build an exon/intron matrix for each read with a custom script (Supplementary methods, Script 1). Reads that matched *ETV4* exons/introns in the same orientation and in the expected proper order were used to reconstruct the structure of the originating transcripts. Finally, aggregated tables containing each reconstructed transcripts with the corresponding number of reads were generated.

The final analysis was limited to the transcripts found with a frequency ≥ 1% in at least one cell line.

### Western blot analysis and subcellular localization

Cells were lysed 24 h after transfection (lysis solution: NaCl 150 mM; Nonidet 40 1%; Sodium deoxycholate 0.5%; SDS 0.1%; Tris–HCl pH8,0 5 mM; EDTA pH8.0 5 mM, PMSF 2 uM; Na3Vo4 1 mM; protease inhibitors cocktail 0,1%); total protein concentration was measured by bicinchoninic acid assay (Thermo Scientific). The proteins were then separated by 10% SDS–PAGE, transferred to PVDF membranes (Bio-Rad), hybridized with primary antibodies and then with horseradish-peroxidase-conjugated secondary antibodies. The signals were detected using Immobilon Crescendo Western HRP Substrate (Merck) and Chemidoc XRS plus (Bio-Rad Hercules, CA, USA). Proteins were detected by the following primary antibodies: *ETV4* Monoclonal Antibody raised against the entire human ETV4 (clone H00002118-M01. Abnova Atlanta, GA, USA) and actin Polyclonal Antibody (clone A2066. Merck). Densitometric quantifications have been performed by using the software ImageJ (https://imagej.nih.gov/ij/).

For the subcellular localization analysis, we obtained nuclear and cytoplasmatic lysates using the NE-PER nuclear and cytoplasmic extraction reagent (Life Technologies) according to the manufacturer's protocol. The lysates were analyzed, as described above, with the monoclonal antibody for ETV4 and, as loading control, monoclonal antibodies for the nuclear Fibrillarin (clone sc-374022. Santa Cruz Biotechnology, Heidelberg, Germany) and for the cytoplasmic HSP90 (clone sc-13119. Santa Cruz Biotechnology).

### Cell Proliferation by MTT assay

Cells were seeded on 96 well plates; cell viability was assessed by the colorimetric assay method based on Thiazolyl Blue Tetrazolium Blue (MTT) (Merck). MTT reagent was added to the cells at a concentration of 0.2 mg/mL. The cells were incubated at 37 °C for 4 h. Crystallized MTT was then dissolved in acidified isopropanol and following the absorbance at 570 nm was measured using Victor X5 Plate Reader (Perkin Elemer, Milan, Italy). After background reading subtraction, the data were expressed as percentage of initial cell number. The experiments were performed 3 times in triplicate.

### Transfection and Dual luciferase reporter assays

The RWPE cells were cultured in 12-well plates to about 60% confluence before transfection. The cells were transfected with plasmids containing cDNA from the full-length or the splice variants of *ETV4* in combination with a plasmid containing part of the promoter of two known targets of ETV4 upstream the firefly gene: *TWIST1*^57^ or *CDKN1A* (p21)^35^. A 845 bp fragment that includes the proximal ETV4 binding site at -676/-671 from transcriptional start site has been used for *CDKN1A*^35^ and a 761 bp fragment that includes the putative ETV4 binding sites at −125/−120; −239/−232 and −261/−256 from transcriptional start site has been used for *TWIST1*^57^. We compared the relative luciferase units (R.L.U.) of each variant to that obtained by ETV4-FL. Cells were also co-transfected with Renilla luciferase pRL-TK reporter vector (Promega, Madison, WI, USA) (ratio 10:1) to normalize luciferase activity. The cells were transfected with the X-tremeGENE HP DNA transfection reagent (Merck) and, after 24 h, were harvested and lysed. Luciferase activity was quantified on the cell lysate using the Dual-Glo Luciferase Assay System (Promega) and the GloMax 20/20 Luminometer (Promega). We calculated the relative luciferase units or R.L.U (firefly/renilla) of each variant and we compared the R.L.U. of each variant to that obtained with ETV4-FL.

### Analysis of ETV4 transcripts in patients with prostate carcinoma (TCGA dataset)

We have selected from the TCGA dataset (https://www.cancer.gov/tcga) samples from patients with prostate cancer for which RNA-seq data were available and that expressed *ETV4* (n = 551). Among these samples we have further selected prostate tumor samples with *ETV4* expression above the 95th percentile (n = 28) and all the peritumoral prostate tissue samples expressing *ETV4* (n = 52). The aligned sequences were downloaded for the analysis. Reads encompassing non-canonical splicing from *ETV4* RNA-seq data have been selected and counted by visual inspection using the “Integrative Genomics Viewer (IGV)”^71,74^.

### Statistical analysis

All data are expressed as mean ± sem. Student t-test was performed using GraphPad Prism v.5.0 for Windows (GraphPad Software, La Jolla, California, USA). Statistical significance was accepted for *P* ≤ 0.05.

## Supplementary Information

Supplementary Information.

## Data Availability

Nanopore sequencing data have been uploaded to the NCBI SRA database (BioProject PRJNA890000) and are available at the following link: https://www.ncbi.nlm.nih.gov/sra/PRJNA890000.
