# Supplementary material for: Identification and characterization of novel ETV4 splice variants in prostate cancer
Source: Sci Rep. 2023 Mar 31;13:5267. doi: 10.1038/s41598-023-29484-1 (PMC10066307; doi:10.1038/s41598-023-29484-1)

Supplementary materials

Identification and characterization of novel ETV4 splice variants in prostate cancer

Irene Cosi [1,2], Annalisa Moccia [1], Chiara Pescucci [1], Uday Munagala [1], Salvatore Di Giorgio [1], Irene Sineo [1], Silvestro G. Conticello [1,3], Rosario Notaro [1,3], Maria De Angioletti [1,2]

[1] Core Research Laboratory, Istituto per lo Studio, la Prevenzione e la Rete Oncologica (ISPRO), Florence, Italy.

[2] ICCOM - National Research Council, Sesto Fiorentino, Florence, Italy.

[3] IFC - National Research Council, Pisa, Italy.

Corresponding author:

Maria De Angioletti, Core Research Laboratory, Istituto per lo Studio, la Prevenzione e la Rete Oncologica (ISPRO), viale Pieraccini 6, 50139 Florence, Italy.

Phone. +39 055 7944584.

e.mail: m.deangioletti@ispro.toscana.it; maria.deangioletti@iccom.cnr.it

Supplementary Results

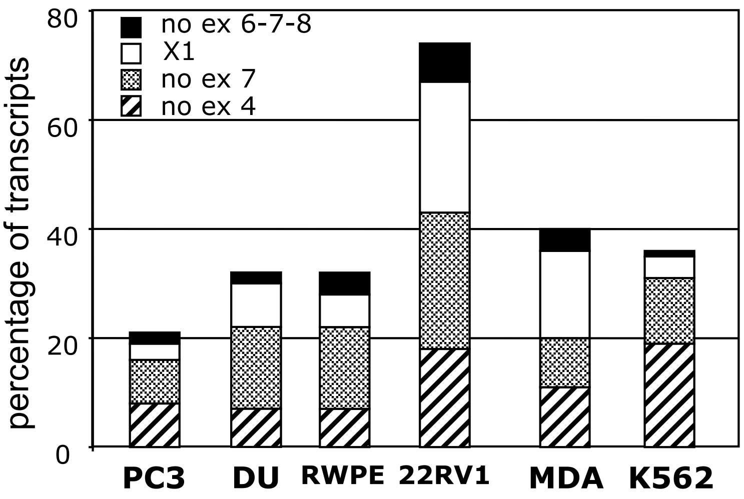

Supplementary Figure 1. Percentage of each *ETV4* splice variant in the respect of total *ETV4* transcripts in the analyzed cells lines.

The expression of each *ETV4* splice variant was measured by RT-qPCR and normalized to the levels of the reference gene *GAPDH* mRNA (*n*=3). Rough quantification of the relative expression of each variant with respect to the total *ETV4* transcripts was done normalizing the expression of each splice variant to the expression of a *ETV4* fragment (exon 11 to exon 12) included in all known splice variants that serves as a proxy control for total *ETV4* transcripts.

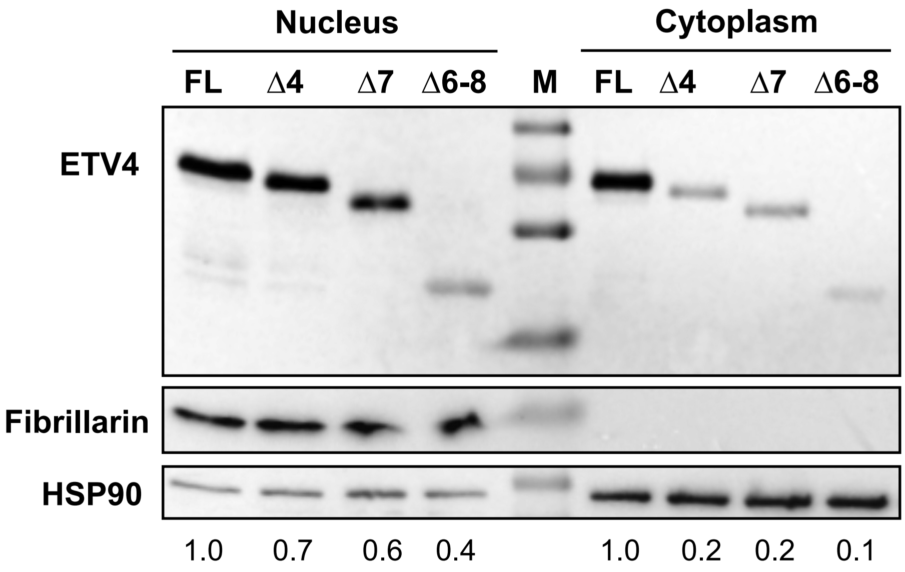

Supplementary Figure 2. Cytoplasm and nuclear localization of proteins translated from splice variants in the MCF7 breast cancer cell line.

Expression of the ETV4 isoforms in the nucleus (left) and the cytoplasm (right) of cells transiently transfected with the indicated ETV4 isoforms (Δ4, Δ7 and Δ6-8) compared with the full-length ETV4 (FL). Fibrillarin and HSP90 have been used as loading control for nucleus and cytoplasm, respectively. The relative ratio of the densitometric quantification of each variant compared with the full-length ETV4 protein is reported. M: molecular weight marker.

Supplementary Methods

mRNA	*Forward primer sequence (primer localization)*	*reverse primer sequence (primer localization)*	*Amplicon Size, bp*	*Method*
ETV4 FL	5'-GCCGCCCCTCGACTCTGAA-3'<br>(Exon 3)	5'-CTGGCCGGTTCTTCTGGATGC-3'<br>(Exon 12)	*1031	RT-PCR
		5'-AGTGGGACAAAGGGACTGTG-3'<br>(Exon 13)	*1241	Cloning
GAPDH	5'-AACGGATTTGGTCGTATTGGGC-3'	5'-TTGATTTTGGAGGGATCTCG-3'	232	RT-PCR<br>RT-qPCR
ETV4 ‡T1, T4, T6	5'-CCCGGCTCCTGGGAGCAG-3'<br>(5' UTR)	5'-TGGAAATCAGGAACAAACTGC-3'<br>(Exon 5)	395 (T6)<br>^314 (T1)<br>203 (T4)	RT-PCR
ETV4 T2, T3	5'-TCCAGCCCAGACGCCCCGGC-3'<br>(5' UTR)		321 (T2)<br>210 (T3)	RT-PCR
ETV4 T5	5'-GGTGAACTTTCCCTGGGATT-3'<br>(5' UTR)	5'-CCTTCCTGCTTGATGTCTCC-3'<br>(Exon 11)	332 (T5)	RT-PCR
ETV4 FL, Δ4	5'-GCCGCCCCTCGACTCTGAA-3'<br>(Exon 3)	5'-GGCACTGGAGTAAAGGCACT-3'<br>(Junction of Exons 6 and 7)	253 (FL)<br>205 (Δ4)	RT-PCR
ETV4 FL, Δ6-8	5'-GCTCGCTGAAGCTCAGGT-3'<br>(Junction of Exons 4 and 5)	5'-CCTTCCTGCTTGATGTCTCC-3'<br>(Exon 11)	**783 (FL)<br>228 (Δ6-8)	RT-PCR
ETV4 FL, Δ7	5'-ACCACCAGGATCAAGAAGGA-3'<br>(Exon 6)	5'-CGAAGTCCGTCTGTTCCTGT-3'<br>(Exon 8)	526 (FL)<br>364 (Δ7)	RT-PCR
ETV4 FL, X2		5'-CGCAGAGGTTTCTCATAGCC-3'<br>(Exon 10)	**637 (FL)<br>371 (X2)	RT-PCR
$ETV4	5'-CGGGCCGGGGAATGGAGT-3'<br>(Exon 11)	5'-TCTGCATGATGCCTTTCTCA-3'<br>(Exon 12)	141	RT-qPCR
ETV4 Δ4	5'-TGAAAGCCGGATACTTGGAC-3'<br>(Exon 2)	5'-GGTACCTGAGCTTCAGAGTCG-3'<br>(Junction of Exon 3 with Exon 5)	151	RT-qPCR
ETV4 Δ7	5'-TTACTCCAGCTCCGTCTTCC-3'<br>(Junction of Exon 6 with Exon 8)	5'-AAAGCTCTGCTGGGGATAGG-3'<br>(Exon 8)	139	RT-qPCR
ETV4 Δ6-8	5'-CATTCAGAAAACCATGTCACC-3'<br>(Junction of Exon 5 with Exon 9)	5'-CGCAGAGGTTTCTCATAGCC-3'<br>(Exon 10)	113	RT-qPCR
ETV4 X1	5'-CGGGGAACATAGATGTCACC-3'<br>(Junction of Exon 7 with Exon 9)	5'-CCTTCCTGCTTGATGTCTCC-3'<br>(Exon 11)	175	RT-qPCR

Supplementary Methods: Table 1: Sequence of primers used for RT-PCR, quantitative RT-PCR (RT-qPCR) and cloning.

Sequence and Exon numbering are according to the NC_000017.11 sequence based on the GRCh38.p13 Primary Assembly.

‡: Transcripts (T) are numbered according the NCBI Reference Sequence database.

*: Only the size of the longer RT-PCR amplicon derived from the full-length transcript is reported.

^: The band of the 314 bp may include the PCR products corresponding to transcripts 7 and 8 that differs from transcript 1 for the deletion of few nucleotides.

**: This primer pair may generate additional amplicons corresponding to potential splice variants (see Figures 2D and 3D)

$: This primer pair amplifies a fragment included in all known splice variants. It serves as a proxy control for total ETV4 mRNA.

Script 1

```
#!/usr/bin/perl

use strict;

# Outputs
my $output = '70complete.txt';
my $output_all = '70missingEx3.txt';
my $output_specials = '70missingBothEnds.txt';
my $output_short = '70short.txt';

my $output_strange = '70strange.txt';
my $output_multiple = '70multiple.txt';
my $output_table = '70table.tsv';
my $output_table1 = '70tableALL.tsv';
open (STRANGE,">$output_strange")||die "Can't open $output_strange:
$!";
open (MULTIPLE,">$output_multiple")||die "Can't open $output_multiple:
$!";
open (TABLE,">$output_table")||die "Can't open $output_table: $!";
open (TABLE1,">$output_table1")||die "Can't open $output_table1: $!";

open (OUT,">$output")||die "Can't open $output: $!";
open (SPECIAL,">$output_specials")||die "Can't open $output_specials:
$!";
open (ALL,">$output_all")||die "Can't open $output_all: $!";
open (SHORT,">$output_short")||die "Can't open $output_short: $!";


# my data
my %reads;       # all the read data
my @cells = qw (nb1 nb2 nb3 nb4 nb5 nb6);
my @files = qw (forward intron3 exon4 intron4 exon5 intron5 exon6
intron6 exon7 intron7 exon8 intron8 exon9 intron9 exon10 intron10
exon11 intron11 exon12 intron12 exon13);
my %length = qw (forward 19 intron3 251 exon4 48 intron4 8496 exon5 54
intron5 2440 exon6 127 intron6 510 exon7 162 intron7 247 exon8 266
intron8 2492 exon9 76 intron9 154 exon10 69 intron10 207 exon11 173
intron11 267 exon12 102 intron12 391 exon13 145);
my %order;   # the order of exons for all reads starting on e3 and
ending on e13
my %order1;   # the order of exons for all reads
my $fraction = 0.5;

#load reads names
print "reading FASTQ file...\n";
open (FASTQ,"total.fastq")||die "Can't open total.fastq: $!";
while (my $line=<FASTQ>){
    if ($line=~/^@(.+) runid=.+$/){
        my $read = $1;
        $reads{$read}{pos}=0;                   # I will add here the
position of the matches
        $reads{$read}{cells}= "no";
        $reads{$read}{orientation}= "no";
        $reads{$read}{length}= 0;
        $reads{$read}{forward} = "no";
        $reads{$read}{intron3} = "no";
        $reads{$read}{exon4} = "no";
```

```
        $reads{$read}{intron4} = "no";
        $reads{$read}{exon5} = "no";
        $reads{$read}{intron5} = "no";
        $reads{$read}{exon6} = "no";
        $reads{$read}{intron6} = "no";
        $reads{$read}{exon7} = "no";
        $reads{$read}{intron7} = "no";
        $reads{$read}{exon8} = "no";
        $reads{$read}{intron8} = "no";
        $reads{$read}{exon9} = "no";
        $reads{$read}{intron9} = "no";
        $reads{$read}{exon10} = "no";
        $reads{$read}{intron10} = "no";
        $reads{$read}{exon11} = "no";
        $reads{$read}{intron11} = "no";
        $reads{$read}{exon12} = "no";
        $reads{$read}{intron12} = "no";
        $reads{$read}{exon13} = "no";
        $reads{$read}{strange} = '';
        $reads{$read}{multiple} = '';
    }
}
close (FASTQ) || die "can't close total.fastq: $!";

# find the cell line
print "assigning the barcode...\n";
foreach my $item (@cells){
    my $file = 'myaln_'.$item.'.maf';
    open (FILE,"$file")||die "Can't open $file: $!";
    while (my $line=<FILE>){
        if ($line =~ /^s (.+?) +(\d+) +(\d+) +(.) +(\d+) +.+/){
            my $read = $1;      # read name
            my $pos = $2;       # position of the match on the read
            my $match = $3;     # length of the aligned match
            my $strand = $4;    # orientation of the read
            my $length = $5;    # total length of the read
            if (exists $reads{$read}){
                if ($length-$pos <100 && $match >11){       # given a
barcode length of 24, 12 seems the minimum match
                    if ($reads{$read}{cells} eq 'no'){
                        $reads{$read}{cells} = $item;
                        $reads{$read}{orientation} = $strand;
                        $reads{$read}{length} = $length;
                    }
                    else {
                        print STRANGE
"$item\t$reads{$read}{cells}\t$read\n";
                        $reads{$read}{strange} = 'yes';
                    }
                }
            }
        }
    }
    close (FILE) || die "can't close $file: $!";
}

# assigning the exons/introns
print "assigning the exons/introns...\n";
foreach my $item (@files){
    my $file = 'myaln_'.$item.'.maf';
```

```
    open (FILE,"$file")||die "Can't open $file: $!";
    while (my $line=<FILE>){
        if ($line =~ /^s (.+?) +(\d+) +(\d+) +(.) +(\d+) +.+/){
            my $read = $1;      # read name
            my $pos = $2;       # position of the match on the read
            my $match = $3;     # length of the aligned match
            my $strand = $4;    # orientation of the read
            my $length = $5;    # total length of the read
            if (exists $reads{$read}){
                if ($strand eq $reads{$read}{orientation} && $pos >
$reads{$read}{pos} && $match >11 && $match/$length{$item} >$fraction){
                    if ($reads{$read}{$item} eq 'no'){
                        $reads{$read}{$item} = $pos.'_'.$match;
                        $reads{$read}{pos} = $pos;
                    }
                    else {
                        print MULTIPLE
"$item\t$reads{$read}{$item}\t$read\n";
                        $reads{$read}{multiple} = 'yes';
                    }
                }
            }
        }
    }
    close (FILE) || die "can't close $file: $!";
}

close (STRANGE) || die "can't close $output_strange: $!";
close (MULTIPLE) || die "can't close $output_multiple: $!";

# counters
print "counting...\n";

print OUT
"read\tcells\torder\torientation\tlength\tforward\tintron3\texon4\tintr
on4\texon5\tintron5\texon6\tintron6\texon7\tintron7\texon8\tintron8\tex
on9\tintron9\texon10\tintron10\texon11\tintron11\texon12\tintron12\texo
n13\n";
print SPECIAL
"read\tcells\torder\torientation\tlength\tforward\tintron3\texon4\tintr
on4\texon5\tintron5\texon6\tintron6\texon7\tintron7\texon8\tintron8\tex
on9\tintron9\texon10\tintron10\texon11\tintron11\texon12\tintron12\texo
n13\n";
print ALL
"read\tcells\torder\torientation\tlength\tforward\tintron3\texon4\tintr
on4\texon5\tintron5\texon6\tintron6\texon7\tintron7\texon8\tintron8\tex
on9\tintron9\texon10\tintron10\texon11\tintron11\texon12\tintron12\texo
n13\n";

foreach my $read (sort {$a <=> $b} keys %reads){
    my $read_order;
    $read_order .= "e3" if ($reads{$read}{forward} ne 'no');
    $read_order .= "_i3" if ($reads{$read}{intron3} ne 'no');
    $read_order .= "_e4" if ($reads{$read}{exon4} ne 'no');
    $read_order .= "_i4" if ($reads{$read}{intron4} ne 'no');
    $read_order .= "_e5" if ($reads{$read}{exon5} ne 'no');
    $read_order .= "_i5" if ($reads{$read}{intron5} ne 'no');
    $read_order .= "_e6" if ($reads{$read}{exon6} ne 'no');
    $read_order .= "_i6" if ($reads{$read}{intron6} ne 'no');
    $read_order .= "_e7" if ($reads{$read}{exon7} ne 'no');
```

```
    $read_order .= "_i7" if ($reads{$read}{intron7} ne 'no');
    $read_order .= "_e8" if ($reads{$read}{exon8} ne 'no');
    $read_order .= "_i8" if ($reads{$read}{intron8} ne 'no');
    $read_order .= "_e9" if ($reads{$read}{exon9} ne 'no');
    $read_order .= "_i9" if ($reads{$read}{intron9} ne 'no');
    $read_order .= "_e10" if ($reads{$read}{exon10} ne 'no');
    $read_order .= "_i10" if ($reads{$read}{intron10} ne 'no');
    $read_order .= "_e11" if ($reads{$read}{exon11} ne 'no');
    $read_order .= "_i11" if ($reads{$read}{intron11} ne 'no');
    $read_order .= "_e12" if ($reads{$read}{exon12} ne 'no');
    $read_order .= "_i12" if ($reads{$read}{intron12} ne 'no');
    $read_order .= "_e13" if ($reads{$read}{exon13} ne 'no');

    unless ($reads{$read}{strange} eq 'yes' || $reads{$read}{multiple}
eq 'yes'){
        if ($reads{$read}{forward} ne 'no' && $reads{$read}{cells} ne
'no'){
            print OUT
"$read\t$reads{$read}{cells}\t$read_order\t$reads{$read}{orientation}\t
$reads{$read}{length}\t$reads{$read}{forward}\t$reads{$read}{intron3}\t
$reads{$read}{exon4}\t$reads{$read}{intron4}\t$reads{$read}{exon5}\t$re
ads{$read}{intron5}\t$reads{$read}{exon6}\t$reads{$read}{intron6}\t$rea
ds{$read}{exon7}\t$reads{$read}{intron7}\t$reads{$read}{exon8}\t$reads{
$read}{intron8}\t$reads{$read}{exon9}\t$reads{$read}{intron9}\t$reads{$
read}{exon10}\t$reads{$read}{intron10}\t$reads{$read}{exon11}\t$reads{$
read}{intron11}\t$reads{$read}{exon12}\t$reads{$read}{intron12}\t$reads
{$read}{exon13}\t\n";
            print ALL
"$read\t$reads{$read}{cells}\t$read_order\t$reads{$read}{orientation}\t
$reads{$read}{length}\t$reads{$read}{forward}\t$reads{$read}{intron3}\t
$reads{$read}{exon4}\t$reads{$read}{intron4}\t$reads{$read}{exon5}\t$re
ads{$read}{intron5}\t$reads{$read}{exon6}\t$reads{$read}{intron6}\t$rea
ds{$read}{exon7}\t$reads{$read}{intron7}\t$reads{$read}{exon8}\t$reads{
$read}{intron8}\t$reads{$read}{exon9}\t$reads{$read}{intron9}\t$reads{$
read}{exon10}\t$reads{$read}{intron10}\t$reads{$read}{exon11}\t$reads{$
read}{intron11}\t$reads{$read}{exon12}\t$reads{$read}{intron12}\t$reads
{$read}{exon13}\t\n";
            $order{$read_order}{$reads{$read}{cells}}++;
            $order1{$read_order}{$reads{$read}{cells}}++;
        }
        elsif ($read_order eq ''){
            print SHORT
"$read\t$reads{$read}{cells}\t$read_order\t$reads{$read}{orientation}\t
$reads{$read}{length}\t$reads{$read}{forward}\t$reads{$read}{intron3}\t
$reads{$read}{exon4}\t$reads{$read}{intron4}\t$reads{$read}{exon5}\t$re
ads{$read}{intron5}\t$reads{$read}{exon6}\t$reads{$read}{intron6}\t$rea
ds{$read}{exon7}\t$reads{$read}{intron7}\t$reads{$read}{exon8}\t$reads{
$read}{intron8}\t$reads{$read}{exon9}\t$reads{$read}{intron9}\t$reads{$
read}{exon10}\t$reads{$read}{intron10}\t$reads{$read}{exon11}\t$reads{$
read}{intron11}\t$reads{$read}{exon12}\t$reads{$read}{intron12}\t$reads
{$read}{exon13}\t\n";
        }
        elsif ($reads{$read}{cells} ne 'no'){
            print ALL
"$read\t$reads{$read}{cells}\t$read_order\t$reads{$read}{orientation}\t
$reads{$read}{length}\t$reads{$read}{forward}\t$reads{$read}{intron3}\t
$reads{$read}{exon4}\t$reads{$read}{intron4}\t$reads{$read}{exon5}\t$re
ads{$read}{intron5}\t$reads{$read}{exon6}\t$reads{$read}{intron6}\t$rea
ds{$read}{exon7}\t$reads{$read}{intron7}\t$reads{$read}{exon8}\t$reads{
$read}{intron8}\t$reads{$read}{exon9}\t$reads{$read}{intron9}\t$reads{$
```

```
read}{exon10}\t$reads{$read}{intron10}\t$reads{$read}{exon11}\t$reads{$
read}{intron11}\t$reads{$read}{exon12}\t$reads{$read}{intron12}\t$reads
{$read}{exon13}\t\n";
            $order1{$read_order}{$reads{$read}{cells}}++;
        }
        else {
            print SPECIAL
"$read\t$reads{$read}{cells}\t$read_order\t$reads{$read}{orientation}\t
$reads{$read}{length}\t$reads{$read}{forward}\t$reads{$read}{intron3}\t
$reads{$read}{exon4}\t$reads{$read}{intron4}\t$reads{$read}{exon5}\t$re
ads{$read}{intron5}\t$reads{$read}{exon6}\t$reads{$read}{intron6}\t$rea
ds{$read}{exon7}\t$reads{$read}{intron7}\t$reads{$read}{exon8}\t$reads{
$read}{intron8}\t$reads{$read}{exon9}\t$reads{$read}{intron9}\t$reads{$
read}{exon10}\t$reads{$read}{intron10}\t$reads{$read}{exon11}\t$reads{$
read}{intron11}\t$reads{$read}{exon12}\t$reads{$read}{intron12}\t$reads
{$read}{exon13}\t\n";
        }
    }
}
close (OUT) || die "can't close $output: $!";
close (ALL) || die "can't close $output_all: $!";
close (SHORT) || die "can't close $output_short: $!";
close (SPECIAL) || die "can't close $output_specials: $!";

print TABLE "isoform\tnb1\tnb2\tnb3\tnb4\tnb5\tnb6\n";
foreach my $isoform (sort {$a <=> $b} keys %order){
    print TABLE "$isoform";
    foreach my $cell (@cells){
        if (exists $order{$isoform}{$cell}){
            print TABLE "\t$order{$isoform}{$cell}";
        }
        else {
            print TABLE "\t0";
        }
    }
    print TABLE "\n";
}
close (TABLE) || die "can't close $output_table: $!";

print TABLE1 "isoform\tnb1\tnb2\tnb3\tnb4\tnb5\tnb6\n";
foreach my $isoform (sort {$a <=> $b} keys %order1){
    print TABLE1 "$isoform";
    foreach my $cell (@cells){
        if (exists $order1{$isoform}{$cell}){
            print TABLE1 "\t$order1{$isoform}{$cell}";
        }
        else {
            print TABLE1 "\t0";
        }
    }
    print TABLE1 "\n";
}

close (TABLE1) || die "can't close $output_table1: $!";
```

ORIGINAL GELS AND BLOTS

Figure 1C

Figure 1C, upper left panel (ETV4)

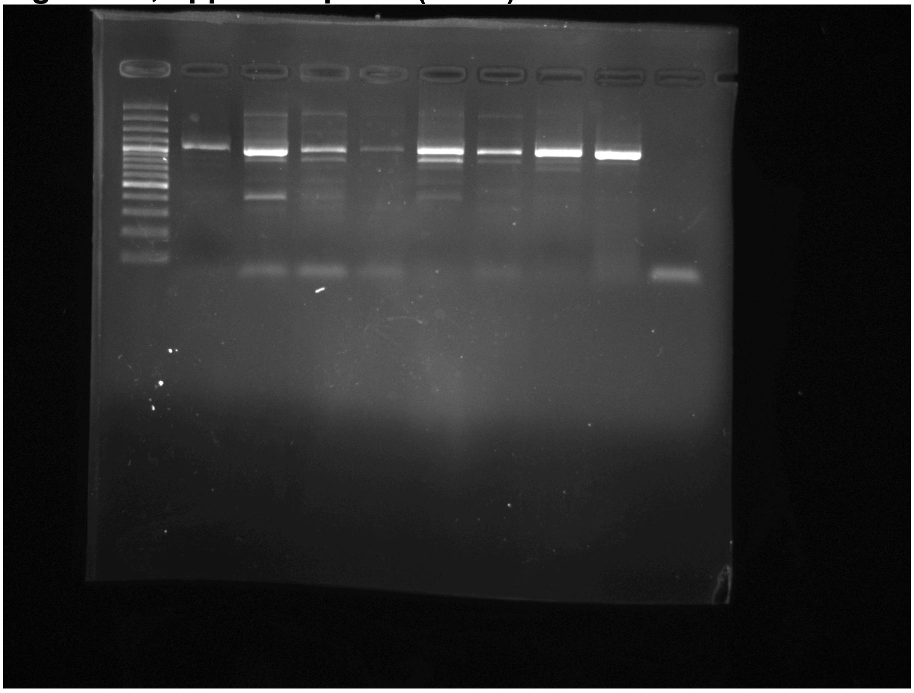

Figure 1C, lower left panel (GAPDH)

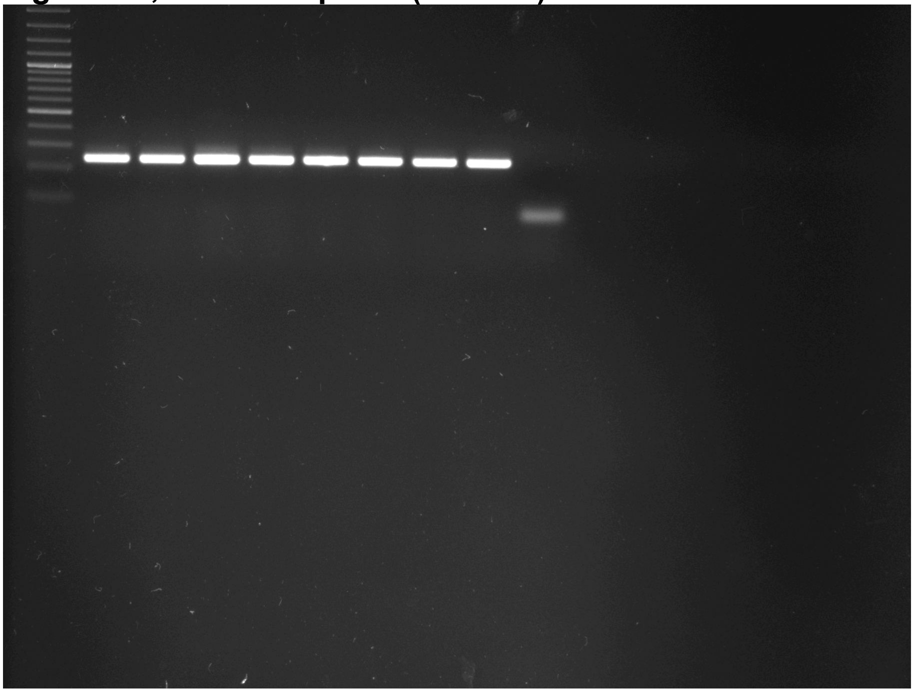

“Identification and characterization of novel ETV4 splice variants in prostate cancer” by Cosi *et al*

Figure 1C, upper right panel (ETV4)

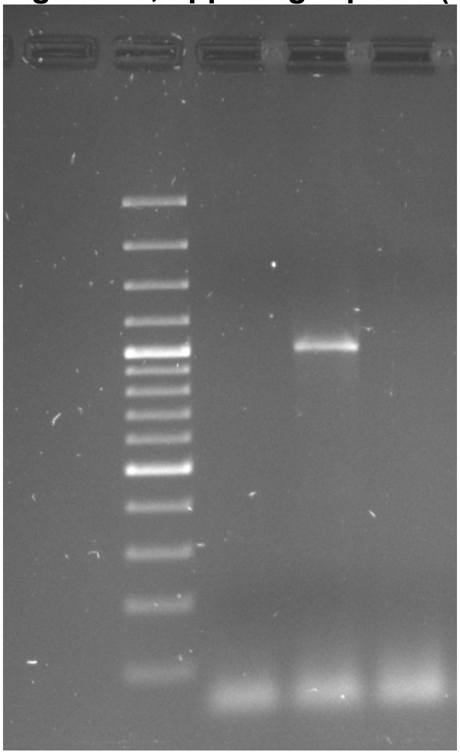

Figure 1C, lower right panel (GAPDH)

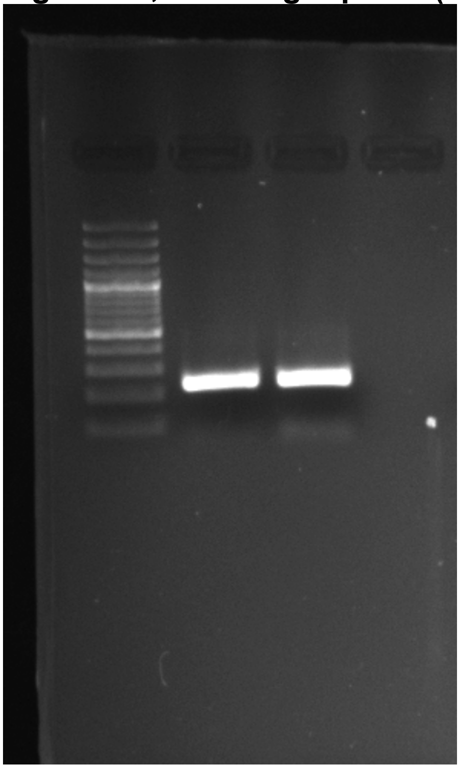

Figure 2

Figure 2A

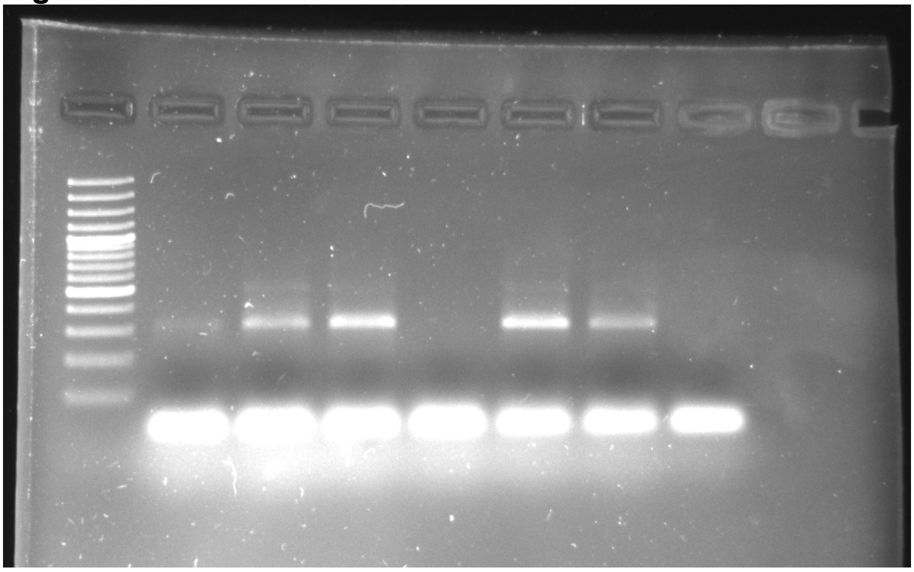

Figure 2B

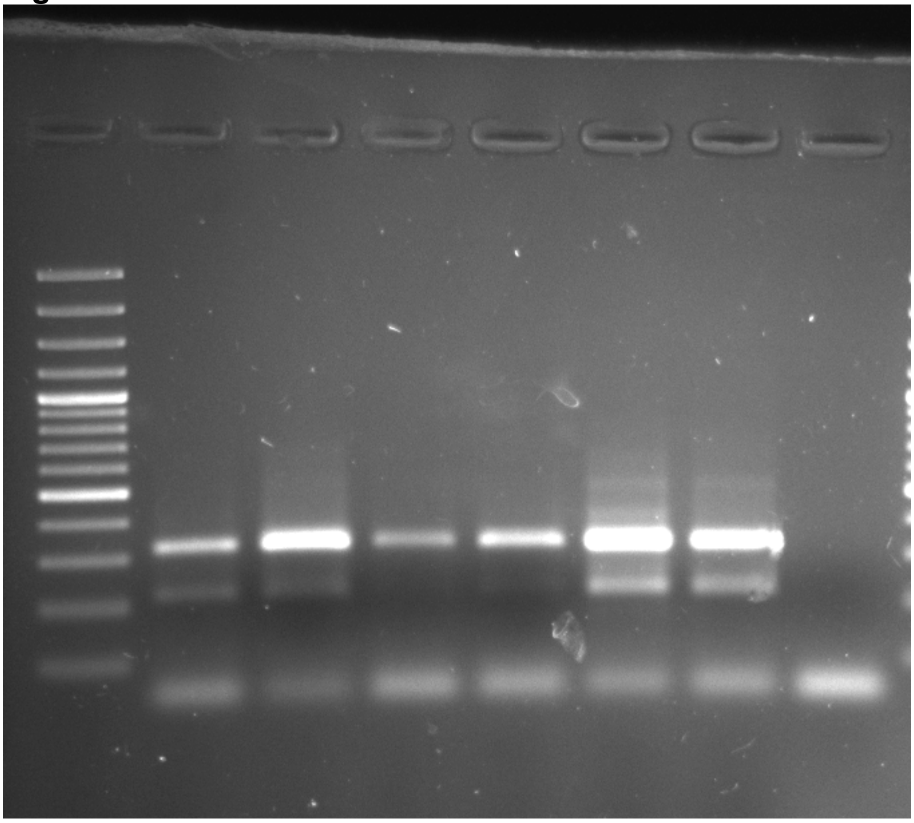

Figure 2C

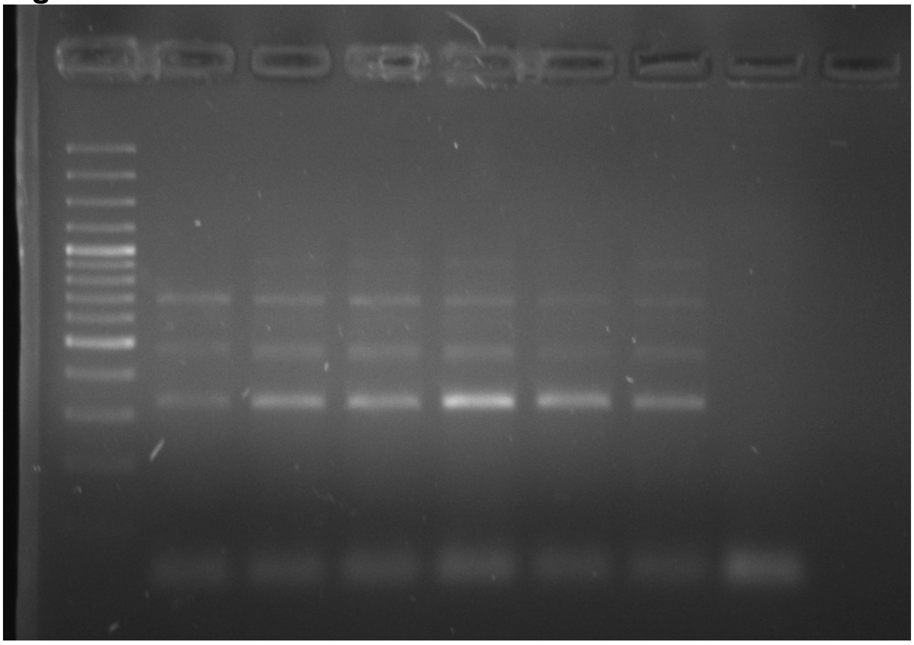

Figure 2D

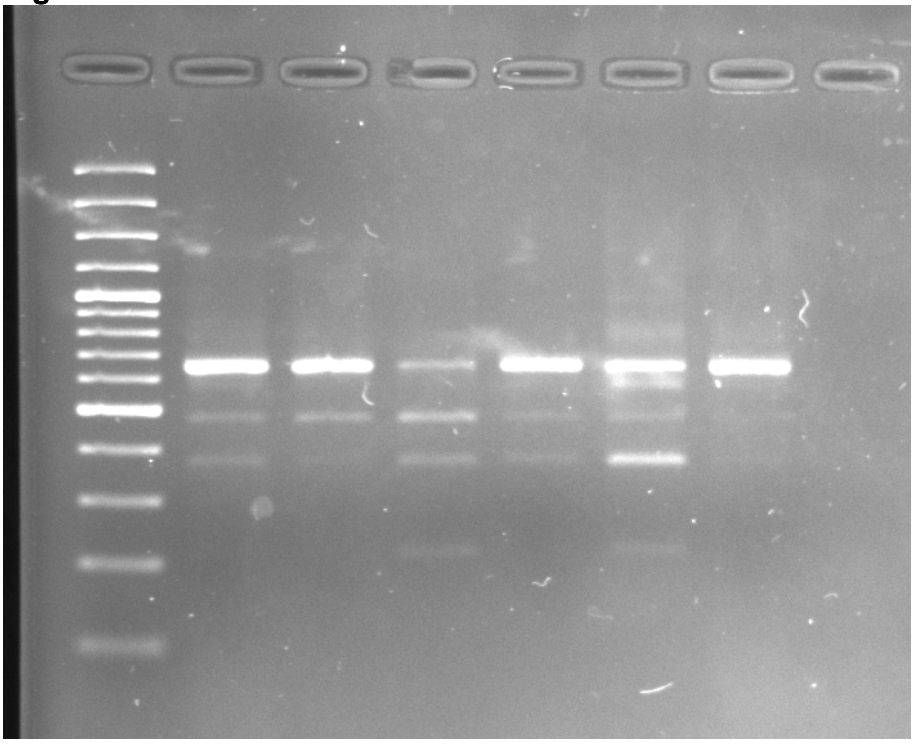

Figure 3

Figure 3B

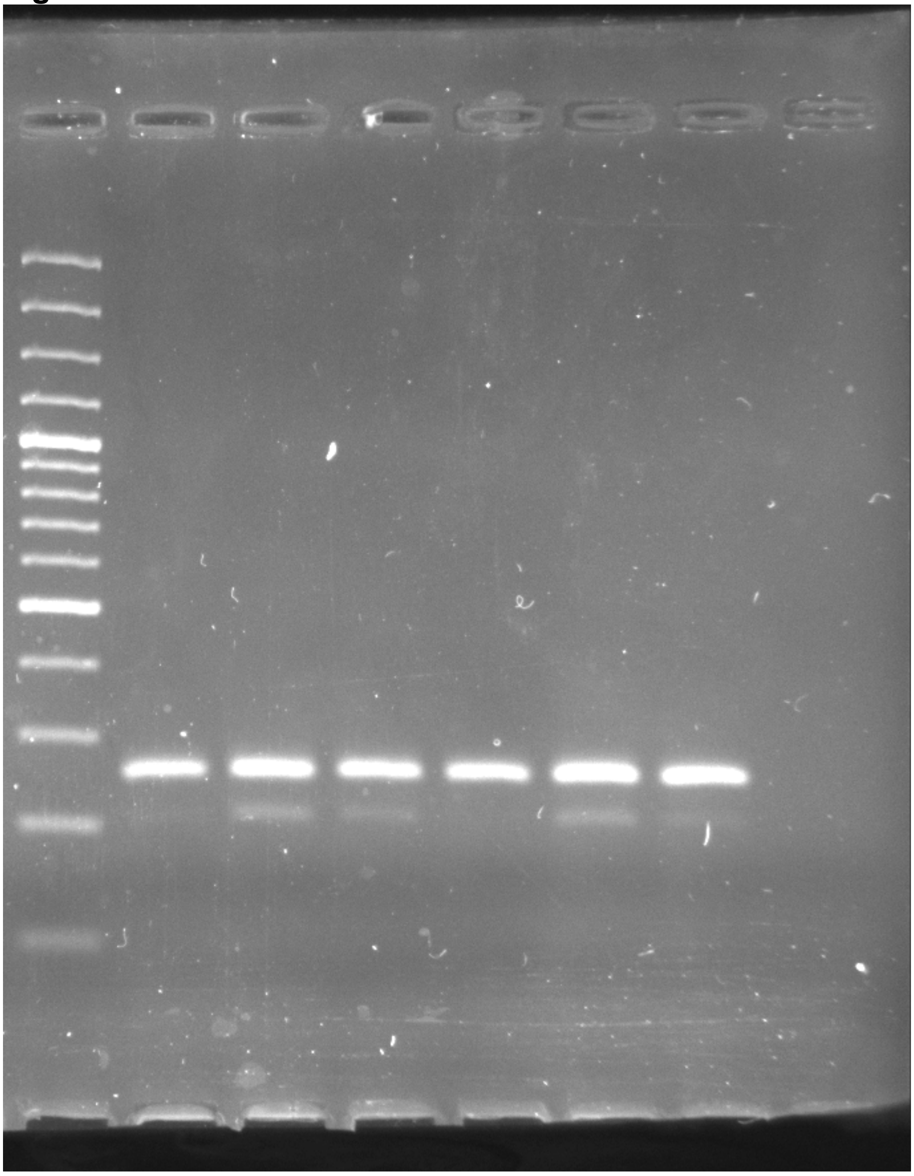

Figure 3C

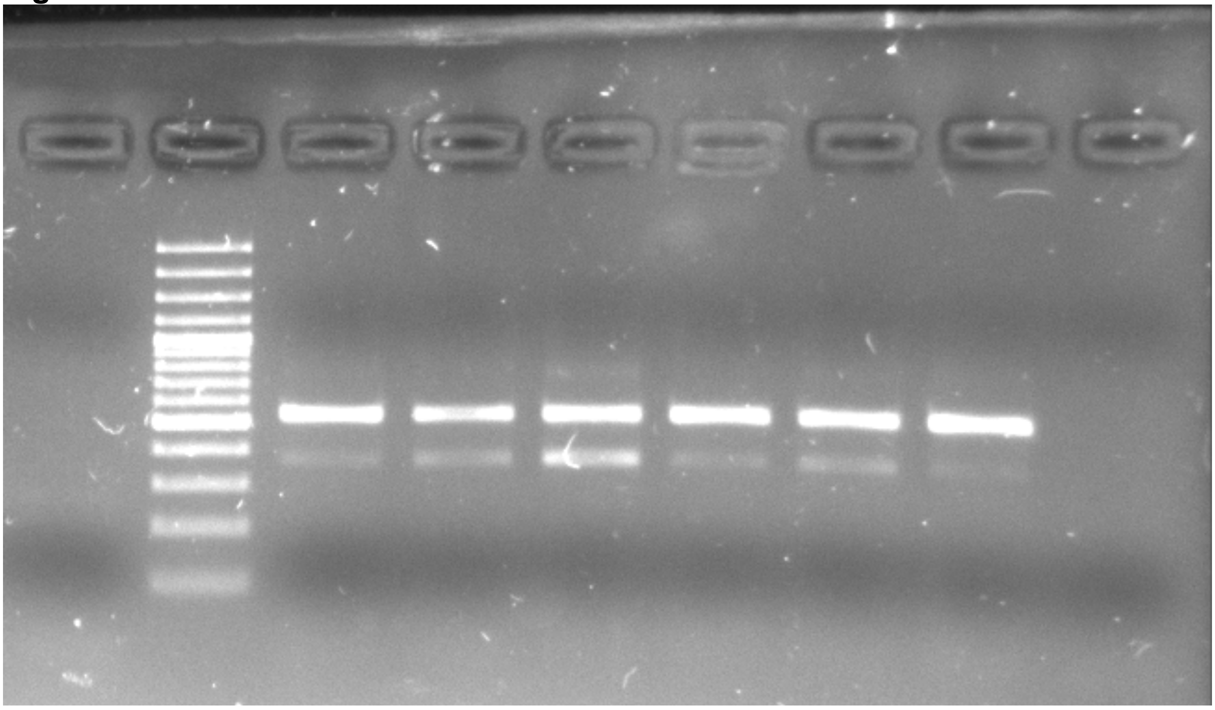

Figure 3D

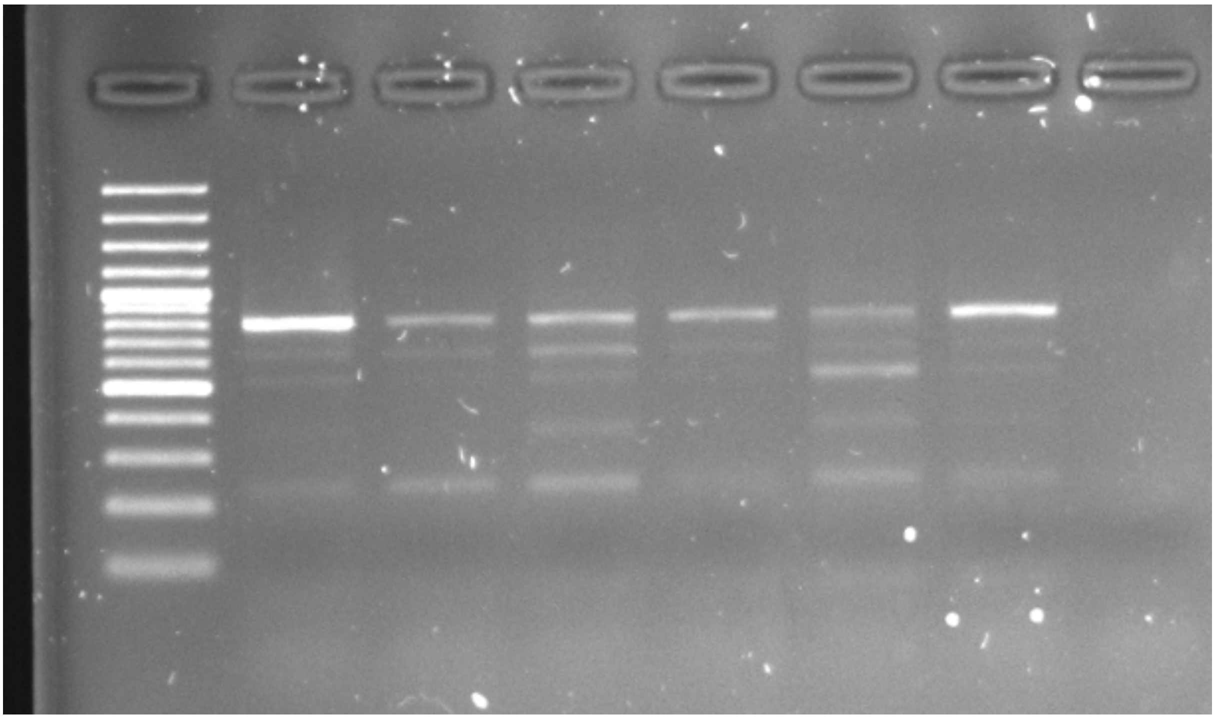

Figure 4 A
These pictures have been enlarged.
Figure 4A left panel, ETV4.tif
Figure 4A left panel, Actin.tif
Figure 4A rigth panel, ETV4 long expos.tif
Figure 4A rigth panel, ETV4 short expos.tif
Figure 4A rigth panel, Actin.tif

Figure 4A upper left panel (ETV4)
Lane 1: FL ETV4
Lane 2: ∆4 variant
Lane 3: ∆7 variant
Lane 4: untransduced control

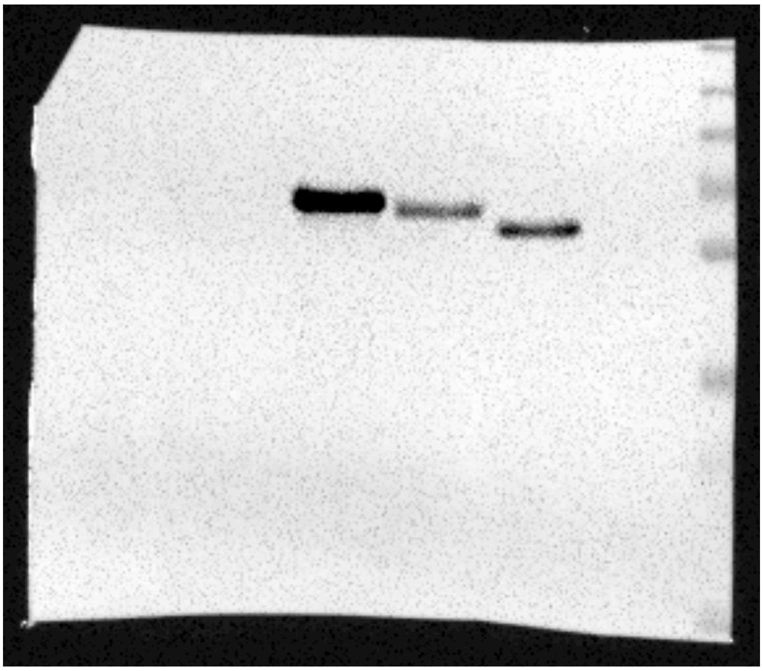

Figure 4A lower left panel (actin)
Lane 1: FL ETV4
Lane 2: ∆4 variant
Lane 3: ∆7 variant
Lane 4: untransduced control

ETV4 from previous hybridization is still visible

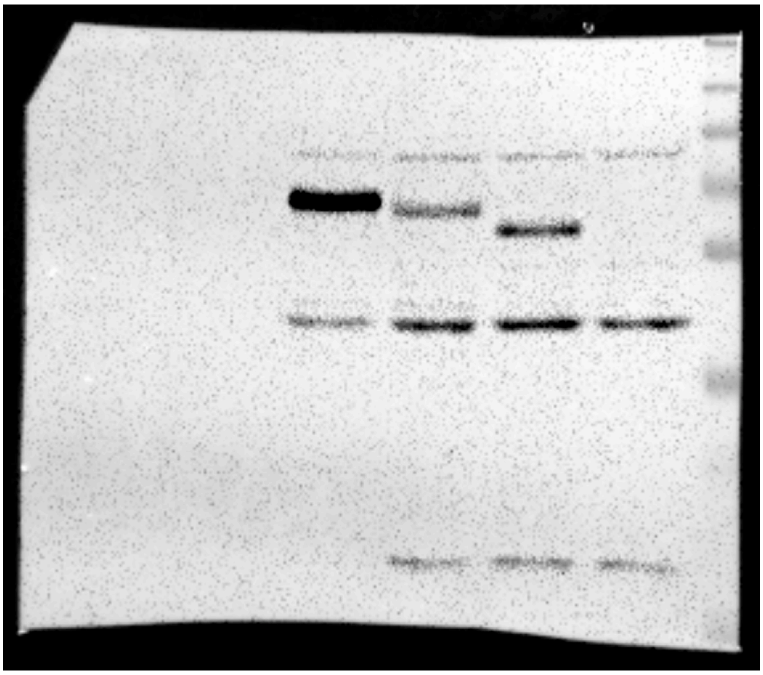

Figure 4A upper right panel (ETV4)
Lane 1: ∆6-8 variant
Lane 2: X1 variant
Lane 3: untransduced control

Short exposure

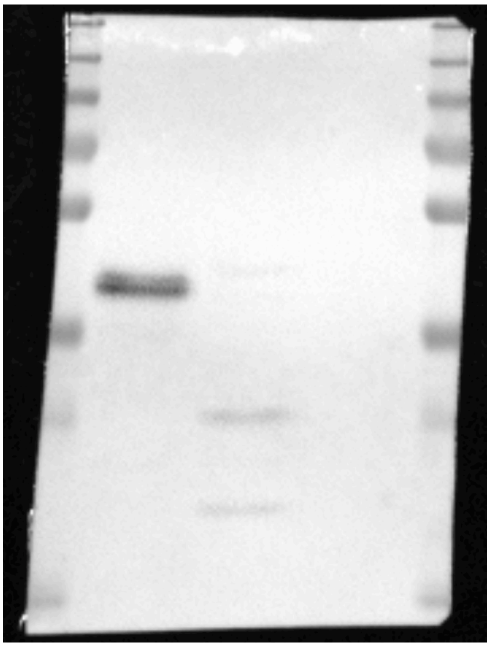

Long exposure
(to show the short X1 variant)

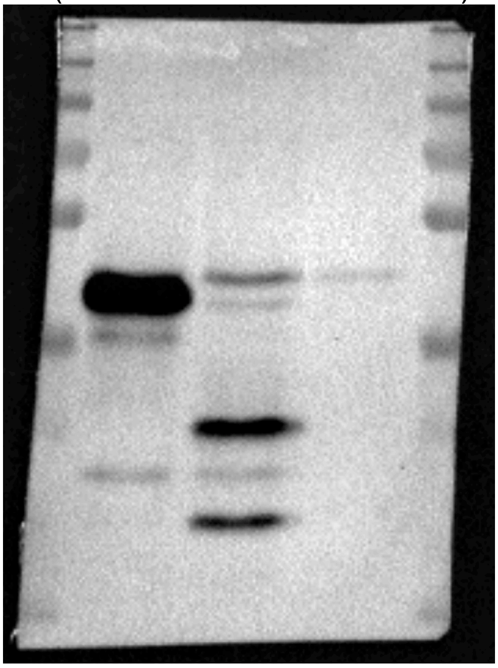

Figure 4A lower right panel (actin)

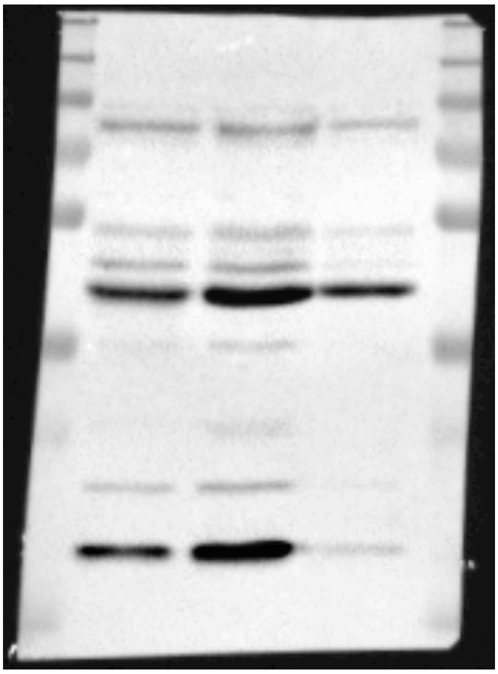

Figure 4 E

Figure 4E (ETV4)
Lane 1 to 4 (nucleus, left), Lane 6 to 9 (cytoplasm, right)
Lane 5: molecular weight marker
Lane 10 control transfected with the empty vector

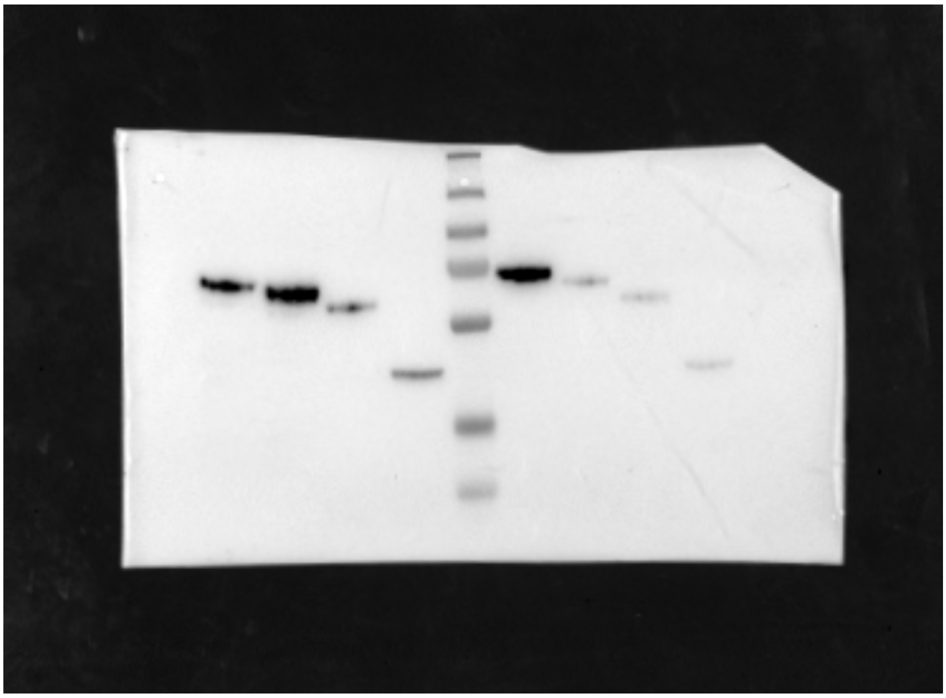

The same gel has been retested with Fibrillarin and HPS90
Lane 5 molecular weight marker
Lane 10 control transfected with the empty vector

Figure 4E (FIBRILLARIN)
Lane 1 to 4 (nucleus, left), Lane 6 to 9 (cytoplasm, right)
ETV4 from previous hybridization is still visible

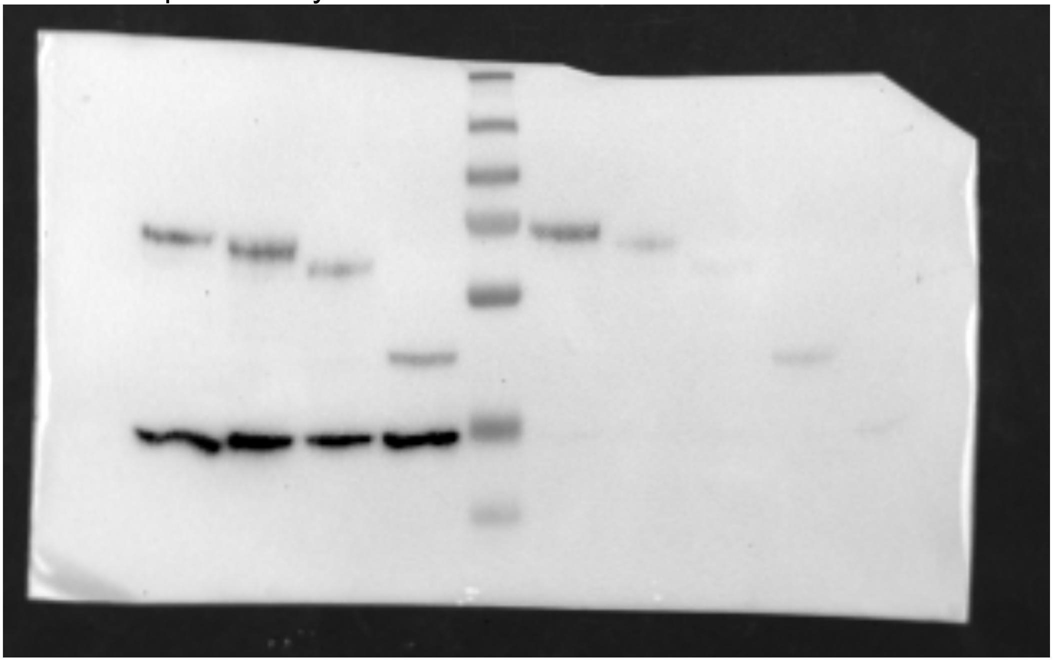

Figure 4E (HSP90)
Lane 1 to 4 (nucleus, left), Lane 6 to 9 (cytoplasm, right)
ETV4 and FIBRILLARIN from previous hybridization are still visible

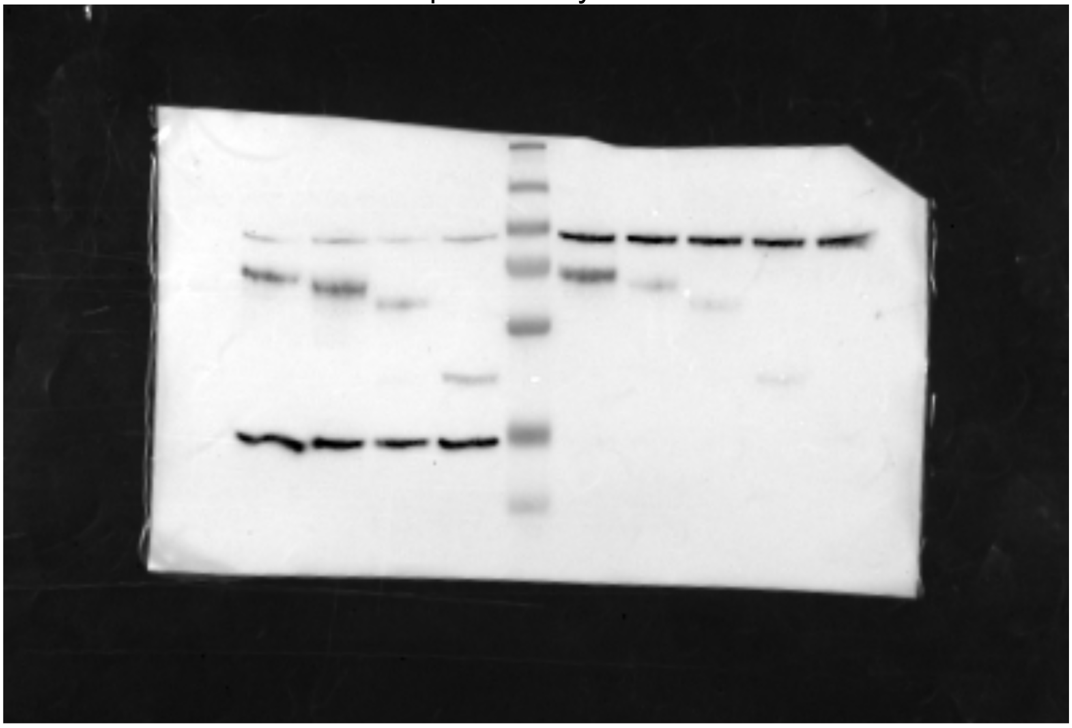

"Identification and characterization of novel ETV4 splice variants in prostate cancer" by Cosi *et al*

Supplementary Figure 2

These pictures have been mirrored to mantain, for the sake of clarity, the NUCLEUS samples on the LEFT.

Suppl Fig2, ETV4.tif
Suppl Fig2, Fibrillarin.tif
Suppl Fig2, HSP90

Supplementary Figure 2
Lane 1 to 4 (nucleus), Lane 6 to 9 (cytoplasm)
Lane 5 molecular weight marker
Lane 10 control transfected with the empty vector

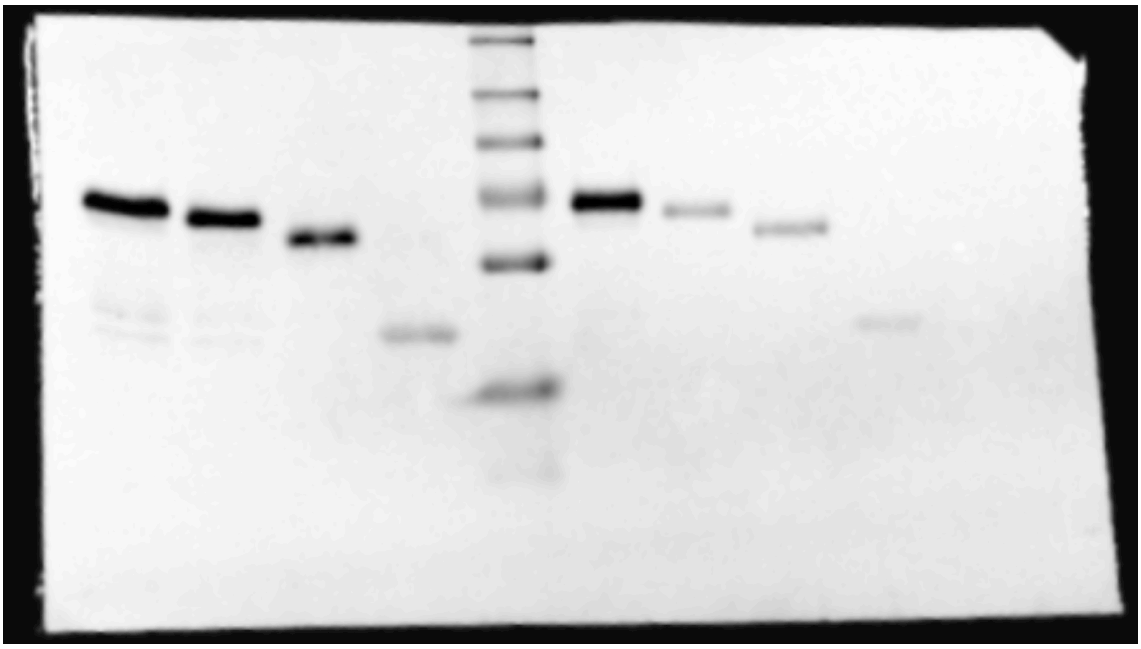

The same gel has been retested with Fibrillarin and HPS90
Lane 5 molecular weight marker
Lane 10 control transfected with the empty vector

Supplementary Figure 2 (FIBRILLARIN)
Lane 1 to 4 (nucleus); Lane 6 to 9 (cytoplasm)
ETV4 from previous hybridization is still visible

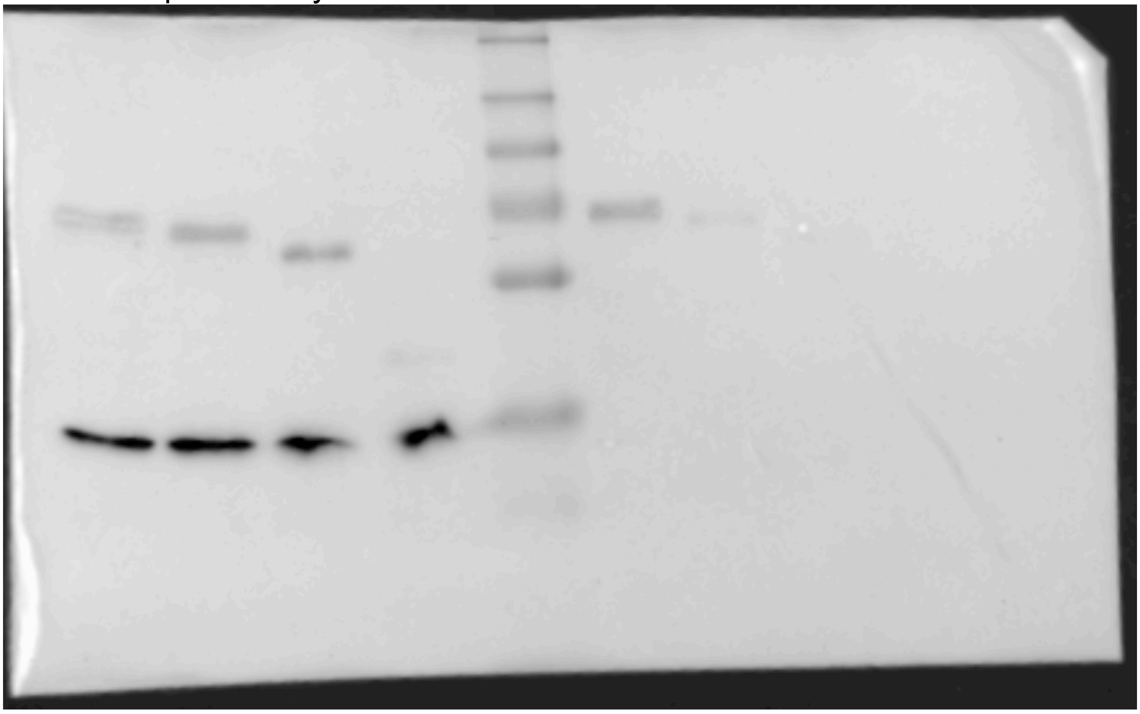

Supplementary Figure 2 (HSP90)
Lane 1 to 4 (nucleus); Lane 6 to 9 (cytoplasm)

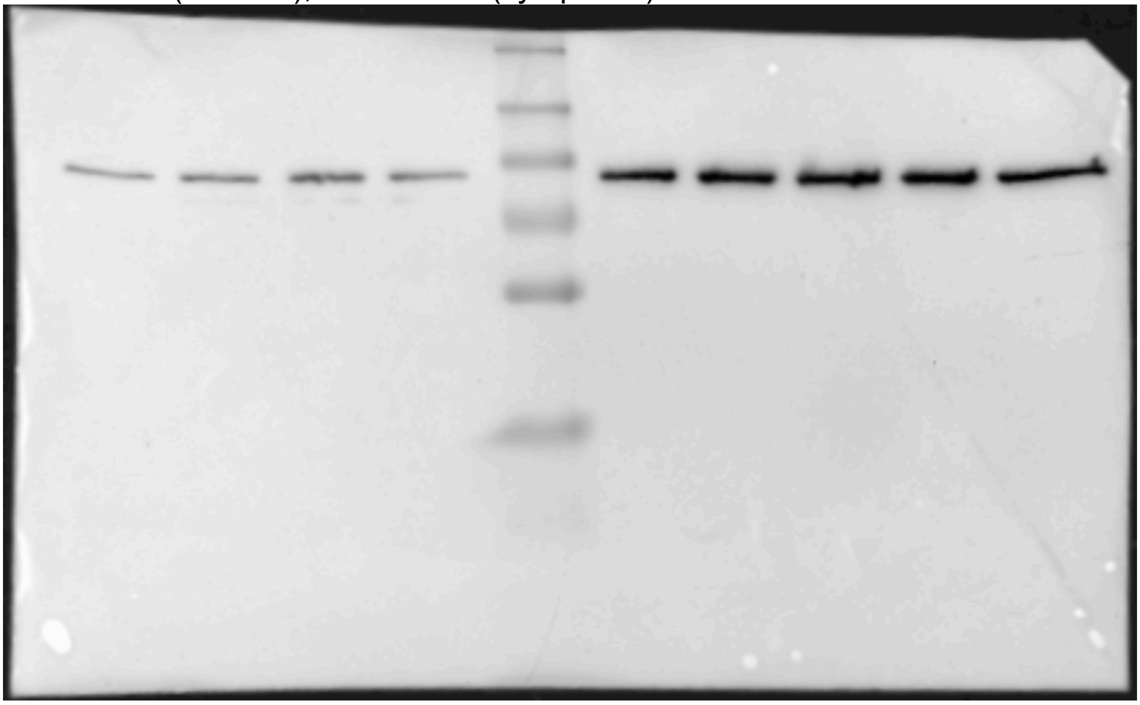

Supplement: Supplementary file 1 — Supplementary Information. [file 41598_2023_29484_MOESM1_ESM.pdf]
